# Programmed cell death in inflammation: interactions and holistic perspective

**DOI:** 10.1038/s41423-026-01479-8

**Published:** 2026-10-13

**Authors:** Wangzheqi Zhang, Chenglong Zhu, Chunzhen Li, Lizhou Song, Yan Liao, Haoling Zhang, Bin Yi, Zui Zou

**Affiliations:** 1 Faculty of Anesthesiology, Changhai Hospital, Naval Medical University, Shanghai, China; 2 School of Anesthesiology, Naval Medical University, Shanghai, China; 3 Department of Anesthesiology, Nanjing Jinling Hospital, Affiliated Hospital of Medical School, Nanjing University, Nanjing, China; 4 National Key Laboratory of Immunity and Inflammation, Naval Medical University, Shanghai, China; 5 Faculty of Naval Medicine, Naval Medical University, Shanghai, China; 6 Department of Biomedical Sciences, Pusat Kanser Tun Abdullah Ahmad Badawi, Universiti Sains Malaysia, Kepala Batas, Malaysia; 7 Department of Anesthesiology, Southwest Hospital, Army Medical University, Chongqing, China

**Keywords:** Programmed cell death, Inflammation, Integrated cell death network, PANoptosis, Cell death crosstalk, Cell biology, Immunology

## Abstract

Programmed cell death (PCD) is a major regulatory mechanism that maintains tissue homeostasis and immune balance and strongly influences the initiation, progression and resolution of inflammation. Although apoptosis, pyroptosis, necroptosis and ferroptosis are individual PCD modes that are regulated by different molecular mechanisms, they are not mutually exclusive in inflammatory microenvironments. Instead, these pathways are linked via common signaling mediators and regulatory nodes that enable crosstalk, compensatory activation and context-dependent fine-tuning. It is evident that apoptosis, pyroptosis and necroptosis can be coordinated within defined inflammatory platforms, as exemplified by concepts such as PANoptosis. Pathway-specific modeling is inadequate for inflammatory diseases involving multiple PCD modalities, metabolic and organelle stress, immune-cell interactions and tissue-level changes that molecular models cannot capture. Here, an integrated cell death network (ICDN) paradigm is presented as an organizational schema describing how various types of PCD are coregulated through common regulatory pathways in inflamed tissues. This framework does not identify a new mode of cell death per se but rather integrates pathway compensation following extracellular perturbations, disease-specific inflammatory responses, and susceptibility to therapeutic intervention at common network nodes. This review defines the molecular mechanisms and crosstalk of inflammatory pyroptosis, apoptosis, and necroptosis pathways; links recent advances in death modality applications for specific diseases to the ICDN framework; and highlights important current limitations that require further examination, including the need for stronger evidence of dynamic switching across cell death modalities, spatial architecture during inflammation, and reconfiguration at both the multiprotein complex and network levels, such as inflammasome networks.

**Figure:**
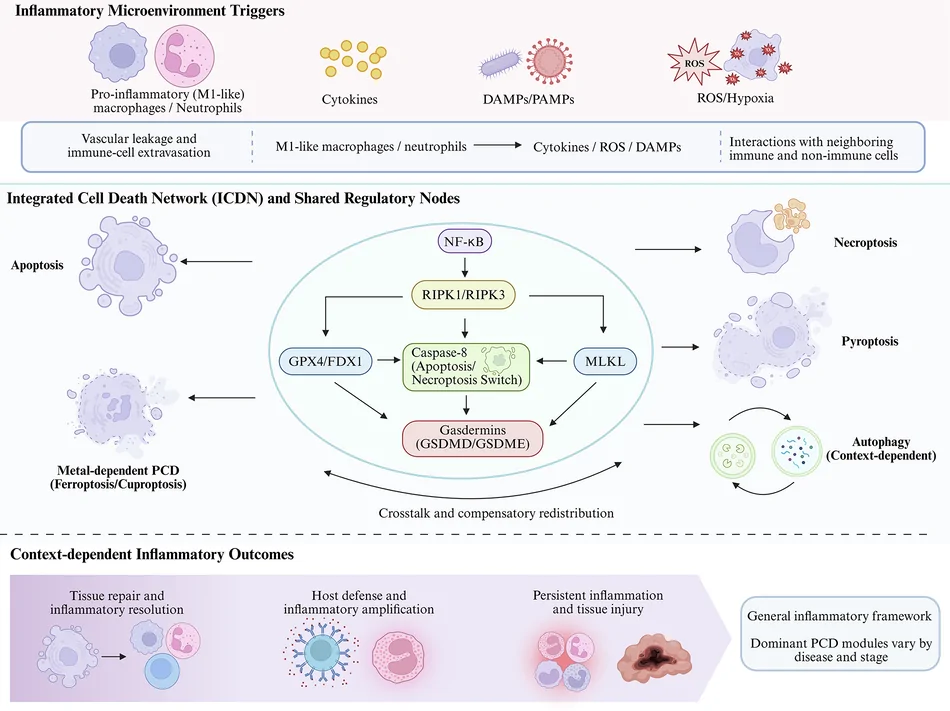

## Introduction

Inflammation is the host reaction to pathogen invasion and tissue damage, but it can also manifest as a persistent state of chronic activation in many diseases, such as infectious diseases, autoimmune diseases, metabolic disorders and cancer [1, 2]. The global burden of morbidity and mortality attributable to inflammation-associated conditions is substantial. For example, chronic inflammation is a key driver of cardiovascular disease, whose global burden of disease is estimated to account for approximately 19.2 million deaths worldwide in 2023, constituting a large proportion of global mortality [3]. Recent advances have highlighted important regulatory roles for cell death in the development of inflammatory responses [4–6]. Distinct modes of cell death are not merely consequences of inflammation; rather, they actively participate in its initiation, progression and resolution by modulating immune cell activation, cytokine release and the extent of tissue injury [7–9]. Accordingly, cell death should not be viewed as a terminal event but as an integral regulatory axis operating throughout the inflammatory continuum.

Cell death can be divided into two major categories, namely, accidental cell death and PCD. The latter consists of a variety of types of cell death, including but not limited to, apoptosis, pyroptosis, necroptosis, autophagy-associated cell death and ferroptosis [10]. Previous studies have primarily distinguished these modes of PCD in terms of their canonical signaling pathways, which has led to major advances that have highlighted the molecular basis by which they operate. However, under inflammatory conditions, cells are subjected not only to cytokine-driven transcriptional signals [11, 12] but also to other danger signals, such as hemodynamic [13] and metabolic signals [14], and multiple forms of PCD may be concurrently engaged or reconfigured during complex inflammatory conditions. This clearly demonstrates that the intracellular programming of inflammatory cell death differs across specific cell types and disease contexts and is coupled with varying gene expression in different tissue microenvironments [12, 15], which cannot be explained using a purely linear, one-pathway model.

While some of these pathways are known to interact, the context-dependent switching between different cell death pathways highlights the necessity of investigating cross-pathway interactions in inflammatory contexts [16]. Emerging studies indicate that various types of PCD are not executed in silos, and the available data suggest that multiple modes of PCD may be orchestrated through common signaling nodes that integrate upstream stimuli to tune the magnitude and nature of inflammatory responses [10, 11, 17]. For instance, gasdermin D (GSDMD) is cleaved by caspase-8 during the course of bacterial infection to initiate pyroptotic execution, indicating that the apoptotic machinery may be redirected toward inflammatory cell death [18]. One more example is the activation of the JAK–STAT1/interferon regulatory factor 1 (IRF1) signaling axis by TNF-α together with IFN-γ to upregulate caspase-8, gasdermin E (GSDME), and mixed lineage kinase domain-like protein (MLKL). Such convergence leads to the processes of apoptosis, pyroptosis, and necroptosis, which are subsequently described as PANoptosis [11]. Finally, PANoptosis is important for understanding the coordinated activation of apoptosis, pyroptosis and necroptosis. However, in addition to these PCD networks, additional death modalities likely exist, together with associated regulatory layers integrating these cell death pathways in inflammatory contexts relevant to diverse pathological states, including ferroptosis, autophagy-associated regulation and metabolic or organelle stress nodes [19]. Accordingly, an ICDN framework is proposed to describe a broader regulatory network in which distinct PCD modalities are coordinated through common molecular conduits and signaling regulated in a context-dependent manner [17, 20, 21]. Importantly, ICDN does not supersede previously described concepts such as PANoptosis or immunogenic cell death (ICD) but rather incorporates them into a broader inflammatory cell death network as execution states or functional consequences.

As a result, this work does not address only isolated varieties of PCD. Instead, it describes the interplay between these pathways in inflammatory conditions, including overlapping regulatory nodes and pathway crosstalk as well as disease-specific inflammatory contexts and novel therapeutic approaches [17, 20]. Moreover, the ICDN framework serves as an organizational and interpretive model rather than a universal mechanism with proven performance. Thus, the present review distinguishes evidence for shared molecular regulators and cross-pathway interactions from more limited direct evidence supporting context-dependent death–modality switching and in vivo coordination among cell death pathways [16, 22].

## PCD pathways and inflammatory mechanisms

**Fig. 1:**
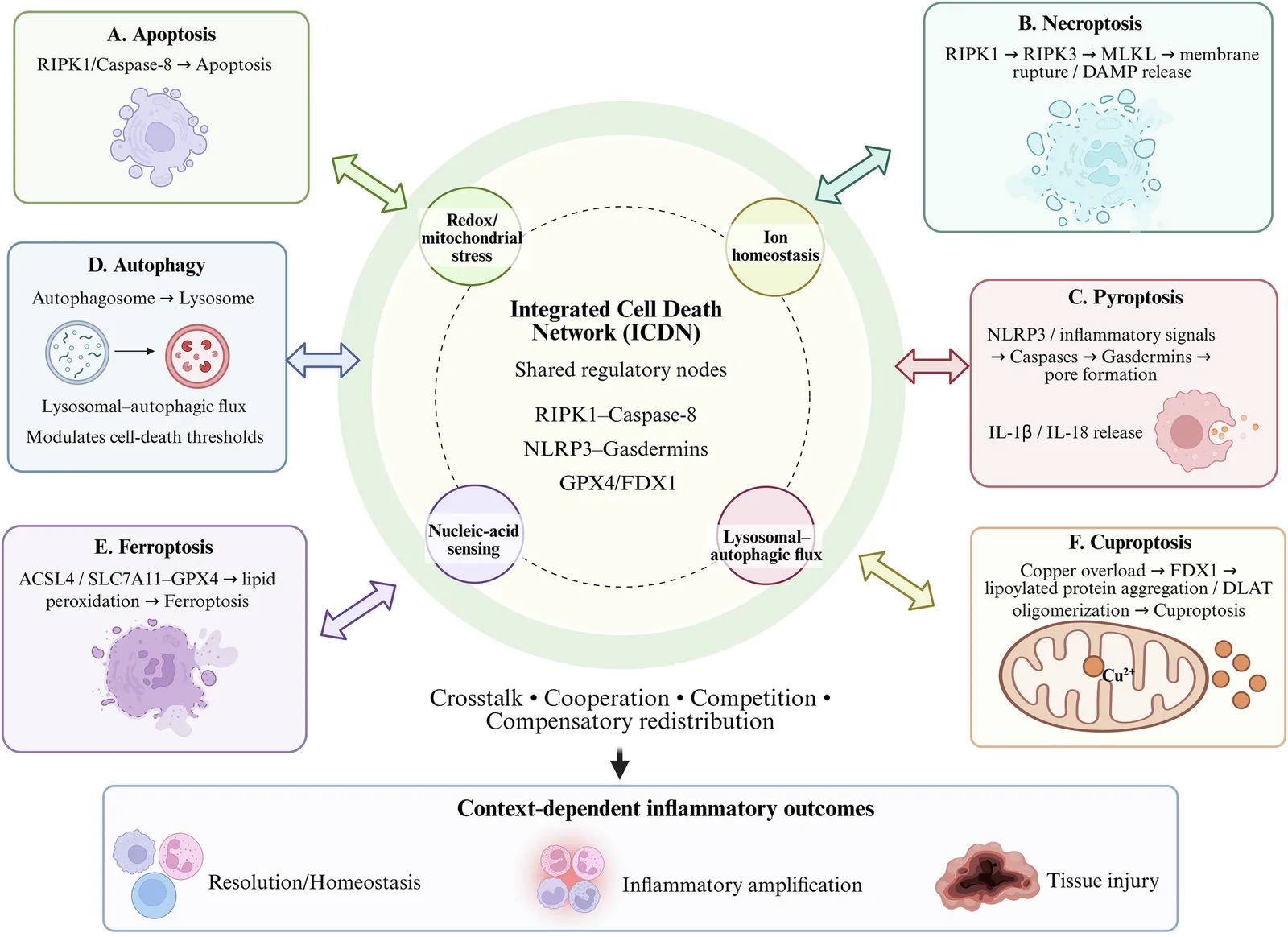
Context-dependent regulatory network of PCD pathways in inflammatory microenvironments. An integrated regulatory network that responds to microenvironmental signals is composed of several PCD pathways within inflamed tissues. Cell death signaling thresholds are modulated by factors such as redox status, ionic homeostasis, nucleic acid sensing intensity and lysosomal-autophagic flux, which shape distinct PCD pathways and their inflammatory outputs. **A** At the cellular level, the RIPK1-caspase-8 complex serves as a major regulator of apoptosis, with either immunologically silent or proinflammatory effects following efferocytosis. **B** Necroptosis occurs through the RIPK1-RIPK3-MLKL signaling axis, resulting in plasma membrane rupture and subsequent DAMP release. **C** Pyroptosis is mediated by gasdermin pore formation, which occurs via the activation of either canonical or noncanonical caspase signaling pathways, resulting in the release of IL-1β and IL-18. **D** In contrast, by removing damaged organelles and inflammatory signaling complexes, autophagy regulates cell death thresholds to modulate several pathways of PCD. **E** Ferroptosis is triggered by ACSL4-mediated lipid peroxidation combined with inactivation of the SLC7A11-GPX4 antioxidant pathway. **F** Cuproptosis occurs when copper accumulates to induce FDX1-dependent aggregation of lipoylated mitochondrial proteins and DLAT oligomerization. Together, these pathways converge at common molecular hubs and inflammatory mediators to create a tightly knit regulatory network that establishes the intensity of inflammatory responses and the degree of tissue damage

In inflammatory microenvironments, unique quanta of PCD deployment do not emanate as compound signal transduction pathways but rather constitute conditional outputs orchestrated by shared regulatory nodes [23]. PCD is seldom triggered via a linear one-pathway system. Rather, it is orchestrated through the coordinated action of myriad intracellular and extracellular determinants, such as redox status, ionic homeostasis and lysosome-autophagy flux, which not only determine the occurrence of cell death but also its modality [24]. These regulatory mechanisms coordinate cellular thresholds for death signaling and shape the differential utilization of pathways. As a result, the same molecular mediators can cluster into context-dependent signaling complexes and produce disparate functional outcomes between tissues or stages of disease, with outputs that may contribute to different phenotypes of cell death as well as inflammatory sequelae [25]. Here, the common molecular machinery and executioner mechanisms of the major PCD pathways are outlined, with a particular focus on conserved regulatory nodes that integrate these pathways. This integrative view offers a framework to interrogate the spatiotemporal distribution of cell death modalities and mechanisms that govern pathway transitions. Under such conditions, different forms of PCD should be seen as alternative outputs in a common regulatory landscape and not as independent processes. The complete signaling network is displayed in Fig. 1, and the evidence and possible therapeutic approaches are summarized in Table 1.

**Table 1:** Major PCD modalities under inflammatory conditions: molecular features and regulatory targets

Cell death modality	Common inflammatory triggers	Shared regulatory nodes and hallmark events	Functional role in inflammation	Relevant cell types or pathological contexts	Potential therapeutic targets and intervention strategies	References
Extrinsic apoptosis (death receptor-mediated)	TNF-α stimulation	TRAF6-mediated regulation of RIPK1-dependent death complex assembly	Negative regulation of cell death to maintain tissue homeostasis	Embryonic liver development; colorectal cancer (CRC) cells	Targeting TRAF6 to sensitize tumor cells to death stimuli	[220]
Extrinsic apoptosis (death receptor-mediated)	Mycoplasma ovipneumoniae infection	Caspase-8 activation with p53/ROS-associated apoptotic signaling	Promotes pro-inflammatory cytokine expression	Murine alveolar macrophages	Inhibitors targeting caspase-8, p53 and ROS	[221]
Extrinsic apoptosis (death receptor-mediated)	Ulcerative colitis and chemically induced inflammation	AKR1B1-mediated activation of NF-κB inflammatory signaling	Promotes inflammation- and oxidative stress-driven tumorigenesis	Colonic epithelial cells; colitis-associated cancer	AKR1B1 inhibitors (e.g., NARI-29)	[222]
Intrinsic apoptosis (mitochondrial pathway)	Ischemia–reperfusion-induced inflammation	Tgm2–HSP70 regulation of ASK1–JNK stress signaling	Reduces neutrophil infiltration and cytokine release	Renal tubular epithelial cells; acute kidney injury	Glutamine targeting Tgm2 and ASK1 pathways	[223]
Intrinsic apoptosis (mitochondrial pathway)	Hypoxia/ischemia-associated inflammation	HIF-1α-associated mitochondrial apoptotic signaling involving Bax, cytochrome c release and caspase-3 activation	Hypoxia-induced epithelial apoptosis leading to bladder injury	Human urothelial cells; interstitial cystitis	Platelet lysate and N-acetylcysteine	[224]
Intrinsic apoptosis (mitochondrial pathway)	TNF-α stimulation	Mitochondrial ROS accumulation leading to caspase-3-dependent apoptosis	Exacerbates endothelial dysfunction and oxidative injury	Endothelial cells; atherosclerosis models	miR-27b and its downstream target FOXO1	[225]
Necroptosis	Age-associated TNF-α elevation and oxidative stress	RIPK1/RIPK3 activation and MLKL phosphorylation-mediated membrane disruption	Release of DAMPs (e.g., HMGB1) drives chronic inflammation	Hepatocytes and macrophages in aged liver; chronic liver disease	RIPK1 inhibitors (e.g., Nec-1 s)	[226]
Necroptosis	Acetaminophen (APAP) overdose-induced oxidative stress and inflammation	PTEN–STING regulation of RIPK3 activation and MLKL phosphorylation	Release of TNF-α and other mediators amplifies inflammation and hepatocyte death	Hepatocytes; APAP-induced acute liver injury	STING inhibitors; RIPK3/MLKL pathway inhibitors	[227]
Necroptosis	TL1A/DR3 axis activation and ovalbumin challenge	RIPK3 activation and MLKL phosphorylation during necroptotic execution	Disrupts epithelial barrier integrity and promotes inflammatory mediator release	Airway epithelial cells; allergic asthma	RIPK3 inhibitor GSK872; targeting MLKL	[228]
Pyroptosis	Bacterial infection and PAMPs/DAMPs	NLRP3 inflammasome activation leading to caspase-1-dependent GSDMD cleavage	Release of IL-1β and other pro-inflammatory cytokines	Macrophages; sepsis and colitis	Bergapten inhibits inflammasome via mitophagy induction	[229]
Pyroptosis	Monosodium urate (MSU) crystals and PAMPs/DAMPs	ROS/thioredoxin-interacting protein (TXNIP)-associated NLRP3 inflammasome activation and GSDMD-mediated pore formation	Drives inflammation through the release of IL-1β and other pro-inflammatory cytokines	Macrophages; gouty arthritis	Corilagin inhibits the ROS/TXNIP/NLRP3 pathway	[230]
Pyroptosis	NETs	AIM2 inflammasome activation with GSDMD cleavage and pyroptotic execution	Promotes inflammatory fibrosis through IL-1β release	Macrophages; chronic inflammatory liver fibrosis	NET degradation or inhibition of the AIM2 inflammasome	[231]
Pyroptosis	Cerebral ischemia/reperfusion injury	LCN2/24p3R-mediated activation of NLRP3 inflammasome signaling	Exacerbates neuroinflammation through the release of IL-1β and IL-18	Astrocytes in cerebral ischemia/reperfusion injury	Inhibition of LCN2 or NLRP3 to attenuate pyroptosis	[232]
Pyroptosis	Aβ plaque deposition	NLRP3 inflammasome activation and GSDMD-dependent inflammatory cell death	Causes neuronal injury through the release of IL-1β and IL-18	Microglia and astrocytes in the AD brain	Inhibition of GSDMD pore formation or NLRP3 activation	[233]
Ferroptosis	Oxygen–glucose deprivation/reoxygenation induced by cerebral ischemia/reperfusion	Iron accumulation, GPX4 inactivation and lipid peroxidation-driven ferroptotic execution	Aggravates neuroinflammation through the accumulation of ROS and lipid peroxides	Microglia in cerebral ischemic injury	Inhibition of the bone morphogenetic protein 6 (BMP6)/SMADs pathway or modulation of iron metabolism	[234]
Ferroptosis	Sepsis	NFIL3-mediated ACSL4 induction promoting lipid peroxidation susceptibility	Exacerbates inflammatory injury through lipid peroxide accumulation	Renal tubular epithelial cells in sepsis-associated kidney injury	Inhibition of the NFIL3–ACSL4 axis	[87]
Ferroptosis	DSS or TNF-α stimulation	cGAS–STING-associated regulation of ferroptosis through GPX4/FTH1 suppression and COX-2 induction	Aggravates epithelial injury and inflammation; suppression of this process is protective	Colonic epithelial cells; DSS-induced colitis in mice	The SP/NK1R axis and the downstream cGAS–STING pathway	[235]
Autophagy	cecal ligation and puncture (CLP)- or LPS-induced acute lung injury/inflammation	NRF2-regulated autophagic machinery involving LC3, Beclin1 and p62	Promotes M2 macrophage polarization, suppresses M1 polarization and alleviates inflammation	Macrophages; CLP-induced septic acute lung injury	The NRF2 signaling pathway and NRF2-mediated autophagy	[236]
Autophagy	Cerebral ischemia/reperfusion injury	Altered autophagic flux reflected by LC3 processing and p62 degradation	Excessive autophagy exacerbates inflammatory injury	Microglia and neurons	Activation of the AKT/mTOR pathway to suppress autophagy	[237]
PANoptosis	Sepsis-associated systemic inflammation	ZBP1/IRF1-associated integration of caspase, gasdermin and RIPK3 signaling pathways	Integrates apoptosis, pyroptosis and necroptosis, thereby amplifying inflammation and influencing prognosis	Peripheral immune cells (such as monocytes and B cells) and tissues from sepsis	Targeting PANoptosis-related genes or immunoregulatory pathways, such as PD-L1	[238]
PANoptosis	Ischemia/reperfusion, hypoxia/reoxygenation and DAMP release	ZBP1-driven PANoptosome assembly involving RIPK3, CASP8, CASP6 and gasdermin-associated pathways	Coordinates apoptosis, pyroptosis and necroptosis to amplify inflammatory injury	Cardiomyocytes (ischemia/reperfusion injury)	The ZBP1 inhibitor MSB and PANoptosome-targeted intervention	[239]

### Apoptosis and inflammation

Although apoptosis normally limits the spread of inflammation, this anti-inflammatory role is based on the proper execution of the apoptotic program and successful clearance of dying cells [26]. In vivo, apoptotic cells retain membrane integrity; therefore, the release of cellular contents and damage-associated molecular patterns (DAMPs) is limited, thus suppressing proinflammatory activity. These cells are quickly identified and phagocytized by phagocytes, which subsequently stimulate anti-inflammatory signaling, promoting tissue homeostasis and the resolution of inflammation [27]. Consequently, apoptosis is often considered an immunological restraint and inhibitor of inflammation [28]. Importantly, the immunological quiescence attributed to apoptosis is predicated on intact effector execution and becomes brittle in complex inflammatory environments.

The immunological effects of apoptosis are highly context-dependent under pathological conditions. Different stimuli, such as infection, oxidative stress and cytokine signaling, can modulate apoptotic signaling, with subsequent changes in inflammatory outcomes. Signaling via death receptors (FasL or TNF-related apoptosis-inducing ligand [TRAIL]) assembles complexes containing receptor-interacting serine/threonine-protein kinase 1 (RIPK1), Fas-associated death domain protein (FADD) and caspase-8, which mediate the initiation of apoptosis but have also been shown to activate NF-κB signaling, promoting the expression of inflammatory genes [29, 30]. Permeabilization of the mitochondrial outer membrane can additionally result in the cytosolic release of mitochondrial RNA, thereby activating the MDA5-MAVS-IRF3 axis and type I interferon responses. Caspase-3 and caspase-7 partially inhibit this process [31]. With the impaired activity of executioner caspases, this regulatory constraint is lost, and apoptotic inflammatory signals are amplified. Under infectious conditions, apoptotic cell clearance may be affected. It has been shown that infection-associated apoptotic cells are engulfed by macrophages, which leads to enhanced production of proinflammatory cytokines and a decreased capacity for long-term clearance [32]. Together, these findings demonstrate that apoptosis is not inherently associated with a low-inflammatory state but instead demonstrates functional plasticity.

Mechanistically, crosstalk between apoptosis and inflammation occurs at highly regulated nodes, including those involved in caspase-8, RIPK1, and NF-κB signaling. For example, caspase-8 can both drive apoptosis and antagonize RIPK1/receptor-interacting serine/threonine-protein kinase 3 (RIPK3)-dependent necroptosis, while in specific contexts, supporting NF-κB-dependent inflammatory signaling [29, 33, 34]. These molecules play a gatekeeping role between death execution and inflammatory signaling and play context-dependent roles [33, 35]. However, the profiles of these components have been investigated mostly within defined pathway frameworks in which their dynamic roles in complex inflammatory environments have not been continuously observed. Moreover, variability in the immunological outcomes of apoptosis makes cross-study comparisons challenging [36]. These limitations highlight the need for a systems-level view. Within this framework, apoptosis can be interpreted as one of several possible outputs regulated by shared nodes such as caspase-8 and RIPK1 rather than as an entirely independent execution pathway. This may occur through context-dependent signaling flux through shared nodes such as caspase-8 and RIPK1 rather than through fixed pathway activation.

Importantly, apoptosis is not an endpoint in itself. It has overlapping regulatory machinery with other forms of cell death, and under certain inflammatory or stress conditions, changes may occur between apoptosis and alternative death programs [16, 37]. Thus, a singular focus on apoptosis may not fully capture the complexity of the inflammatory processes at play, lending further support to an integrated network-based perspective of cell death. However, direct longitudinal in vivo evidence of real-time transitions between apoptotic and other death states remains limited, and such conclusions are typically deduced from shared regulators and perturbation studies [37].

### Necroptosis and inflammation

Necroptosis is a caspase-independent type of cell death that usually results from the loss of caspase-8 function or dominant-negative regulation of death receptor signaling and relies on the RIPK1-RIPK3-MLKL signal transduction pathway [38]. It is characterized by rupture of the plasma membrane, directly connecting cell death to inflammatory activation [39]. Unlike accidental necrosis resulting from a physical or chemical insult, necroptosis is an active, regulated molecular process rather than a passive fall to cellular ruin. While necroptosis and unregulated necrosis both display common morphological characteristics, such as cellular swelling and cell-membrane rupture, they differ at the regulatory level and have different signaling dependencies [40]. In complex tissue environments, the definitions of necroptosis, secondary necrosis and other lytic death modalities are, however, only incompletely understood.

During the execution phase, necroptosis is generally triggered by upstream signaling via RIPK1 downstream of death receptors, pattern recognition receptors or nucleic acid-sensing pathways [41]. When caspase-8 is inhibited, RIPK1 pairs with RIPK3 to form the necrosome and phosphorylate MLKL. MLKL is activated, oligomerizes, and translocates to the plasma membrane, where it disrupts membrane integrity and causes the release of intracellular contents [42]. This ordered sequence of events, including the activation of RIPK1, RIPK3 and MLKL; plasma membrane rupture; and the release of DAMPs, accounts for not only the programmed nature and proinflammatory potential of necroptosis but also its common placement upstream in inflammatory circuits.

Necroptosis has been widely reported to promote inflammatory responses through DAMP release and immune activation; however, some studies have indicated that its inflammatory contribution varies across cell types and disease models, suggesting that necroptosis may act as either a driver or a secondary amplifier depending on the inflammatory context. The release of intracellular components, including DAMPs and cytosolic proteins, via plasma membrane rupture also contributes to inflammatory signaling [43]. In addition, necroptosis is a critical component of nucleic acid-sensing pathways. For instance, STING-driven Z-DNA binding protein 1 (ZBP1) potentiates RIPK3 activity and drives necroptosis, whereas mitochondrial DNA release can be fed back into the cyclic GMP-AMP synthase (cGAS) and STING pathways, thereby forming a feed-forward inflammatory loop [44–46]. These findings suggest that necroptosis is not merely a downstream consequence of inflammation but can actively contribute to its initiation and propagation. Nevertheless, most studies have focused on DAMP release and cytokine responses, with limited systematic comparisons across cell types and tissue contexts.

Inflammation, in turn, can further promote necroptosis. Cytokines and oxidative stress in the inflammatory microenvironment regulate the activation of the RIPK1, RIPK3 and MLKL axes, thus altering cellular susceptibility to necroptosis [47]. In systemic inflammatory response syndrome, RIPK3–MLKL-dependent necroptosis of endothelial cells occurs upstream of vascular barrier disruption and increases vascular permeability, facilitating the infiltration of inflammatory components, structural tissue changes and injury to organs [39]. Deletion of MLKL or RIPK3 dampens immune cell infiltration and attenuates tissue remodeling in models of aging-associated inflammation [39, 48, 49] and corneal injury. Similarly, inhibition of RIPK3 or MLKL postviral infection mitigates inflammation severity and increases survival [50]. Although the particulars differ across conditions, these studies as a whole suggest an amplifying loop whereby inflammation induces necroptosis and vice versa.

From a therapeutic standpoint, the intersection of necroptosis and inflammation lies at key nodes, including RIPK1, RIPK3, MLKL, and the cGAS–STING pathway. Therefore, these nodes connect cell death with inflammatory signaling, making them potential therapeutic targets [45, 51]. However, more recent evidence has shown that blocking single molecules results in variable results in different models, likely because of the development of compensatory pathways and switching between cell death modalities [42]. RIPK1 is a good example of this complexity; it coordinates multiple forms of cell death, such as apoptosis and necroptosis, as well as inflammatory signaling, and its inhibition can have context-dependent consequences [52]. In accordance with this, therapeutic strategies are moving from single-pathway inhibition to integrated modulation of cell death networks. Novel strategies include the combined targeting of necroptosis and other types of death (such as ferroptosis), as well as the use of selective regulators generated by molecular design and computer approaches.

Most current investigations are still molecular centered, and only a few studies have investigated the spatial distribution of necroptosis among tissues and, inevitably, its association with inflammatory dissemination. Together, the intricate relationship between necroptosis and inflammation cannot be reconciled with a single pathway alone but rather may reflect the integration of distinct forms of cell death mediated via common regulatory mechanisms [11]. Therefore, further studies should include spatial mapping, temporal analysis and in vivo functional validation of its role at different stages of disease to discern optimal windows for intervention [53]. Necroptosis is also linked to apoptosis and pyroptosis within the ICDN through shared regulatory nodes involving RIPK1, RIPK3, and caspase-8. Its activation may, counterintuitively, reflect changes in signaling at these common nodes rather than completely independent pathway engagement [16, 54].

### Pyroptosis and inflammation

Pyroptosis is a type of PCD mediated by the gasdermin family and is characterized by plasma membrane pore formation and proinflammatory mediator release [55, 56]. In most experimental settings, pyroptosis is characterized by gasdermin-mediated membrane pore formation and the release of intracellular contents, leading to strong proinflammatory responses. Unlike apoptosis, a rapid increase in membrane permeability occurs during pyroptosis, and proinflammatory cytokines (such as IL-1β and IL-18) are released [57]. Thus, cell death can turn into an inflammatory signal that spreads throughout the body and significantly amplifies the inflammatory response. Nevertheless, most existing studies are based on a single-cell type model, and systematic comparisons of pyroptotic phenotypes across different cellular backgrounds have not been well characterized.

The initiation of pyroptosis-associated inflammation primarily depends on canonical and noncanonical inflammasome pathways. In the canonical pathway, inflammasomes such as NLR family pyrin domain containing 3 (NLRP3) activate caspase-1, which in turn cleaves GSDMD and promotes the maturation and release of inflammatory cytokines [58]. In the noncanonical pathway, murine caspase-11 or human caspase-4/5 directly sense cytosolic lipopolysaccharide (LPS), triggering GSDMD-dependent inflammatory responses and exacerbating tissue injury in gram-negative bacterial infections and sepsis [59, 60]. Moreover, K⁺ efflux promotes NLRP3 signaling, establishing a positive feedback loop that augments caspase-1 activation and GSDMD cleavage. Pyroptosis can also be triggered by viral infections through similar pathways; GSDMD deficiency ameliorates lung injury in the case of influenza invasion [61]. These mechanistic insights notwithstanding, most studies have focused on upstream inflammasome activation, while the field’s understanding of the propagation of inflammatory signals from tissues where gasdermin pores are opened has not yet fully matured. Therefore, the key deciding factor for pyroptosis is not only inflammasome activation but also whether downstream gasdermin cleavage, pore formation and IL-1β/IL-18 release actually occur to drive inflammatory amplification.

In turn, pyroptosis feeds back to regulate inflammation, resulting in the formation of a bidirectional regulatory axis [62]. Cytokines, reactive oxygen species (ROS) and metabolic changes in the inflammatory microenvironment facilitate inflammasome assembly and promote gasdermin cleavage, thereby increasing the risk of pyroptosis [57, 58]. On the other hand, cells can also restrict pyroptosis-associated inflammatory output via membrane repair mechanisms or through the adoption of different death modalities. Notably, the endosomal sorting complex required for transport III (ESCRT-III)-mediated membrane repair may alleviate the damage caused by GSDMD pores, thus limiting cell lysis and broad inflammatory dissemination [63]. This alternative pathway possibly occurs in specific settings, where cells may shift from pyroptosis to apoptosis, thus leading to the attenuation of inflammation. In neutrophils, the activation of GSDMD drives pore formation and inflammation in a neutrophil extracellular trap (NET)-dependent manner that sustains and amplifies inflammatory signal transduction [64]; however, other studies suggest that NET release can occur independently of GSDMD, indicating that its requirement is context- and stimulus dependent. In contrast, GSDME deficiency skews cell death toward apoptosis and inhibits inflammatory spread through enhanced phagocytic clearance [65]. These findings emphasize that pyroptosis and inflammation occur through a multilayer regulatory network rather than through a linear cascade. However, the temporal interplay of this bidirectional regulation and its disease-specific variability remain underdefined.

Key intervention points for pyroptosis and inflammation coupled from a therapeutic perspective focus on gasdermin pore formation and its downstream regulation. A promising strategy focuses on targeting GSDMD pores; for example, nanobodies can block pore formation without inhibiting GSDMD cleavage and thus reduce inflammatory output without perturbing upstream signaling [66]. Importantly, gasdermin pores appear to be transiently reversible, enabling the regulation of cytokine release. Therefore, some pore-forming cells may escape cell death and continue signaling inflammatory processes [63].

However, current advances are predominantly limited to molecular mechanisms, with few reports addressing the spatial dynamics of pyroptosis within tissues and differential membrane repair capacity and interactions with other cell death modalities. Additionally, discrepancies in the measurement of pyroptosis and inflammatory output between models add further complexity to interpretation. Pyroptosis-driven inflammation is driven not only by inflammasome assembly but also by execution-phase regulation and tissue-level dynamics, suggesting that pyroptotic signaling should be reassessed along an integrative paradigm. In the ICDN, pyroptosis can be seen as a high-efficiency inflammatory output state given the preferential allocation of gasdermin signaling between competing execution modules. It is important to study pyroptosis in this context because it does not hinge only on the activation of an inflammasome; indeed, multiple concurrent death signals might exist, whereby cooperative apoptotic or necroptotic signaling can channel cellular fate and graded inflammatory outcomes.

### Autophagy and inflammation

Autophagy is a lysosome-dependent degradative process involved in the turnover of dysfunctional organelles and proteins [67]. Under inflammatory conditions, autophagy not only mediates the clearance of intracellular constituents but also plays an important role in modulating the duration of inflammatory signaling, with subsequent effects on cellular responses [68]. Its defining role here is twofold. Autophagy not only acts as a recycling machinery for cellular components but also directly controls the persistence and termination of inflammatory signals, making it one of the key determinants of cell fate. In conditions with intact autophagic flux, inflammasome components and damaged organelles are efficiently cleared, and inflammation is maintained within physiological limits [68]. Conversely, impaired autophagy or lysosomal dysfunction leads to their accumulation, which can potentiate inflammatory signaling and promote the transition to PCD [69, 70]. Thus, autophagy not only exerts protective effects but also determines, to some extent, the reversibility of inflammation.

In most environments, autophagy inhibits inflammation by clearing activated inflammatory species and promoting signal resolution. In the inflammasome pathway, NLRP3 not only is regulated at the level of activation but can also be degraded by autophagy [71], which limits the persistence of inflammatory signaling [72]. Autophagy mediated by zinc finger DHHC-type containing 12 (zDHHC12) promotes NLRP3 degradation, suggesting that inflammasome maintenance is actively regulated [73]. In nucleic acid-sensing pathways, STING must be degraded in endolysosomes when activated; otherwise, its vesicular colocalization within the cytosol promotes interferon signaling [74]. This process relies on the ESCRT machinery and is associated with noncanonical autophagy [75]. In addition, nucleotide-binding oligomerization domain-containing protein 1 (NOD1) plays a protective role by specifically removing apoptosis signal-regulating kinase 1 (ASK1) through autophagy-related 16-like 1 (ATG16L1)-dependent autophagy, thereby inhibiting stress-induced inflammatory amplification [76]. Taken together, these findings suggest that autophagy not only represses signal initiation but also, more importantly, guarantees timely termination of inflammatory responses. However, single studies typically explore only a subset of pathways, and integrated coordinated regulation across multiple inflammatory circuits has been poorly defined.

Although autophagy is usually considered anti-inflammatory, it can instead promote inflammation if it is dysregulated in certain contexts [77]. Autophagy can modulate cytokine production and release by affecting inflammasome assembly and activity. In addition, defective autophagic flux causes the accumulation of dysfunctional mitochondria and ROS and sustains inflammatory signaling, reinforcing a proinflammatory environment [78]. In other cases, in particular contexts, autophagy promotes inflammatory cell function through the provision of metabolic substrates. These observations highlight the context-dependent nature of autophagy, which primarily has anti-inflammatory effects, depending on the cell state and microenvironment [68]. Conversely, inflammation also regulates autophagy. Under physiological or mild stress conditions, inflammatory signals can signal autophagy, especially mitophagy, to remove damaged mitochondria and decrease the number of ROS-producing organelles that can threaten cellular homeostasis. In contrast, prolonged or intense stimulation results in a vicious cycle of mitochondrial damage and excessive ROS, which stoke the NLRP3 inflammasome and aggravate the inflammatory response while simultaneously reducing autophagic function [70].

Key regulatory links between autophagy and inflammation include inflammasome activation, selective autophagy receptors (e.g., p62), STING-dependent pathways and lysosomal integrity. Not only do these processes fine-tune inflammatory signaling, but they also dictate the lifetime of autophagic flux. Nevertheless, the present studies are mostly limited from molecular and cellular perspectives [79], with relatively few references to the heterogeneous nature of autophagy within tissues and its flux dynamics. Inconsistent methods for measuring autophagic activity also complicate interpretation [80]. Apart from signaling modulation, autophagy also plays a role in maintaining tissue homeostasis by tuning the amplitude of inflammation and associated cellular states, necessitating more integrated studies across disease contexts [68]. Autophagy regulation is involved mainly in the ICDN model, modulating cell death programs by regulating signal duration and organelle homeostasis.

### Ferroptosis and inflammation

Ferroptosis is an iron-catalyzed form of regulated cell death characterized by the peroxidation of membrane phospholipids enriched in polyunsaturated fatty acids (PUFAs), the depletion of glutathione and the inactivation of glutathione peroxidase 4 (GPX4) [81]. In addition to redox imbalance, ferroptosis is intimately associated with the inflammatory microenvironment, in which lipid peroxidation and oxidative stress contribute to tissue damage and aggravate inflammatory responses, affecting disease progression [82, 83]. Mechanistically, ferroptosis can be viewed as the intersection of three linked processes, including the increased availability of iron, the peroxidative degradation of PUFA-containing phospholipids, and the collapse of the GPX4-based antioxidant defense system.

Acyl-CoA synthetase long-chain family member 4 (ACSL4), which, during execution, regulates PUFA incorporation into membrane phospholipids, sensitizes cells to lipid peroxidation, and helps link lipid metabolic remodeling to inflammatory injury [84, 85]. Despite differences in upstream stimuli (e.g., STING activation, radiation stress and transcriptional reprogramming), many models converge on the upregulation of ACSL4 coupled with increased lipid peroxidation. This dynamic has been observed in various inflammation-linked settings, but the relative role of this axis likely differs markedly across diseases [86–88].

Moreover, disruption of antioxidant systems is critical for determining the threshold involved in ferroptosis and its inflammatory consequences. GPX4, the central suppressor of ferroptosis, is required for immune cell survival and functional homeostasis [89]. Ferroptosis has been linked to the survival of intestinal NKp46⁺ ILC3s, and GPX4 loss reduced this population and inhibited IL-22 and IL-17A production, subsequently compromising mucosal barrier integrity and promoting colitis [90]. These results therefore indicate that ferroptosis indirectly influences the establishment of inflammatory microenvironments by affecting immune homeostasis.

Ferroptosis is often associated with lipid peroxidation and the release of DAMPs that can activate pattern recognition receptors and downstream inflammatory cascades [91]; however, the extent to which ferroptosis directly drives inflammation varies across models, with some studies showing limited or context-dependent inflammatory consequences despite the release of DAMPs [92]. For instance, high mobility group box 1 (HMGB1), which is released upon ferroptosis, interacts with TLR4 on macrophages and activates the STAT3 signaling pathway, which leads to proinflammatory responses and allows the intercellular amplification of inflammation [93]. In addition, fibroblasts can undergo ferroptosis induced by NETs, which inhibit collagen formation and exacerbate the delay in healing in patients with diabetes, suggesting that ferroptosis acts on inflammatory signaling but also affects the outcome of chronic inflammation because of defective tissue repair [94].

Ferroptosis can reciprocally be regulated by inflammation. In inflammatory microenvironments, cytokines, ROS and the excessive formation of free iron products from iron metabolism lead to cell death by ferroptosis. Continued inflammatory stimuli lead to ROS accumulation and inhibit antioxidant systems, thereby compromising GPX4-dependent protection [95]. Simultaneously, inflammatory signaling disrupts iron homeostasis and lipid metabolism, exacerbating lipid peroxidation [96]. These rearrangements make inflammation itself a constitutive permissive condition for ferroptosis. However, systematic comparisons of the effects of individual inflammatory mediators on the regulation of ferroptosis are lacking, and their tissue- and disease-specific actions are poorly defined.

By linking lipid peroxidation, GPX4-dependent antioxidant collapse and DAMP-dependent proinflammatory signaling, ferroptosis closely connects metabolic dysregulation to inflammation [95]. In distinct paradigms of inflammation-mediated tissue damage, ferroptosis arises as a sequela of oxidative stress and lipid remodeling but also serves as an active driver of the inflammatory response through the regulation of immune homeostasis and signal amplification [97]. However, existing studies focus mostly on the one-way influence of ferroptosis on inflammation, while the reciprocal regulation of inflammation and its stage-dependent role are still poorly characterized. Moreover, further studies are warranted to elucidate the tissue-level distribution of ferroptosis and its interaction with other cell death modalities [98]. Underlining the ICDN framework, ferroptosis is linked to apoptosis, pyroptosis and necroptosis through a common redox imbalance and organelle stress. This is expected when global changes in the cellular redox state occur, and it is often correlated with other death modalities that are not necessarily independent pathways but instead reflect similar stress conditions.

### Cuproptosis and inflammation

Cuproptosis is a newly defined form of metal-dependent PCD caused by intracellular copper overload and disruption of mitochondrial metabolism. This process is highly dependent on the upstream regulation of ferredoxin 1 (FDX1), which promotes the aberrant aggregation of lipoylated mitochondrial proteins, particularly dihydrolipoamide S-acetyltransferase (DLAT), and disruption of tricarboxylic acid cycle function [99, 100]. This ultimately leads to proteotoxic stress and, ultimately, cell death [101]. In contrast to other types of PCD, cuproptosis is more directly regulated by metal ion homeostasis and the mitochondrial metabolic state and is thus frequently associated with inflammation-associated tissue injury in the context of metabolic stress [102]. However, current evidence is largely derived from specific experimental systems, and its generalizability and tissue specificity remain to be established.

With respect to inflammatory amplification, cuproptosis might contribute to inflammation through metabolic perturbation and mitochondrial dysfunction. In various models, copper overload is linked to tissue damage and increased inflammation [99, 103]. In cholestasis and colitis, for example, increased copper retention or local accumulation leads to disruption of the lipoylated protein network, which is dependent on FDX1, along with upregulation of DLAT oligomerization and inflammatory mediators [104, 105]. Similar findings have been shown in models of lung injury, in which oxidative stress increases intracellular copper concentrations and induces increased proinflammatory cytokine release [103]. These data imply that cuproptosis is not simply a byproduct of metabolic derangement but can participate in enhancing inflammatory signaling. However, most current studies are still correlative, and causal relationships in the initiation of inflammation have yet to be firmly established.

Conversely, susceptibility to cuproptosis can also be regulated by inflammatory signaling [106]. During inflammatory stress, oxidative stress and mitochondrial dysfunction can be accompanied by disturbed intracellular copper homeostasis and altered expression of copper transporters (e.g., copper transporter 1 [CTR1]) and cuproptosis-related metabolic regulators (e.g., FDX1 and lipoic acid synthase [LIAS]), potentially changing cell sensitivity toward cuproptosis [107, 108]. Inflammatory signaling can also reduce cuproptotic susceptibility through the transcriptional modulation of cuproptosis-related regulators [106]. However, the corresponding molecular mechanisms remain incompletely defined, and their functions across disease stages have not been systematically compared.

Pathologically, the links between cuproptosis and inflammation involve immune cell regulation and cross-talk with other modalities of cell death [109]. SLC31A1-mediated copper accumulation, for instance, promotes macrophage polarization toward a proinflammatory phenotype, which enhances the inflammatory response [110]. Moreover, cuproptosis may mediate ferroptosis by affecting the homeostasis of intracellular metals. Copper overload disrupts redox balance and iron metabolism, leading to enhanced cell sensitivity to ferroptosis [111]. While emerging evidence suggests interplay between metal-dependent cell death pathways, existing interventions are centered primarily around copper chelation and metabolic modulation. Their mechanisms and efficacy in more complex inflammatory environments remain to be elucidated.

In inflammation-initiated tissue injury, cuproptosis can be triggered by the dysregulation of copper homeostasis and the amplification of inflammatory responses, resulting in the reshaping of the immune microenvironment [99]. However, current evidence has emerged mainly from organ-specific models with little cross-tissue or human data. Therefore, the dynamic interplay between inflammation and cuproptosis is not well understood. Under the umbrella of ICDN, cuproptosis connects metal homeostasis with mitochondrial metabolism to provide a model for how metal-dependent events can modulate the dynamic regulation of cell death modalities during inflammation.

## Crosstalk in the PCD network

**Fig. 2:**
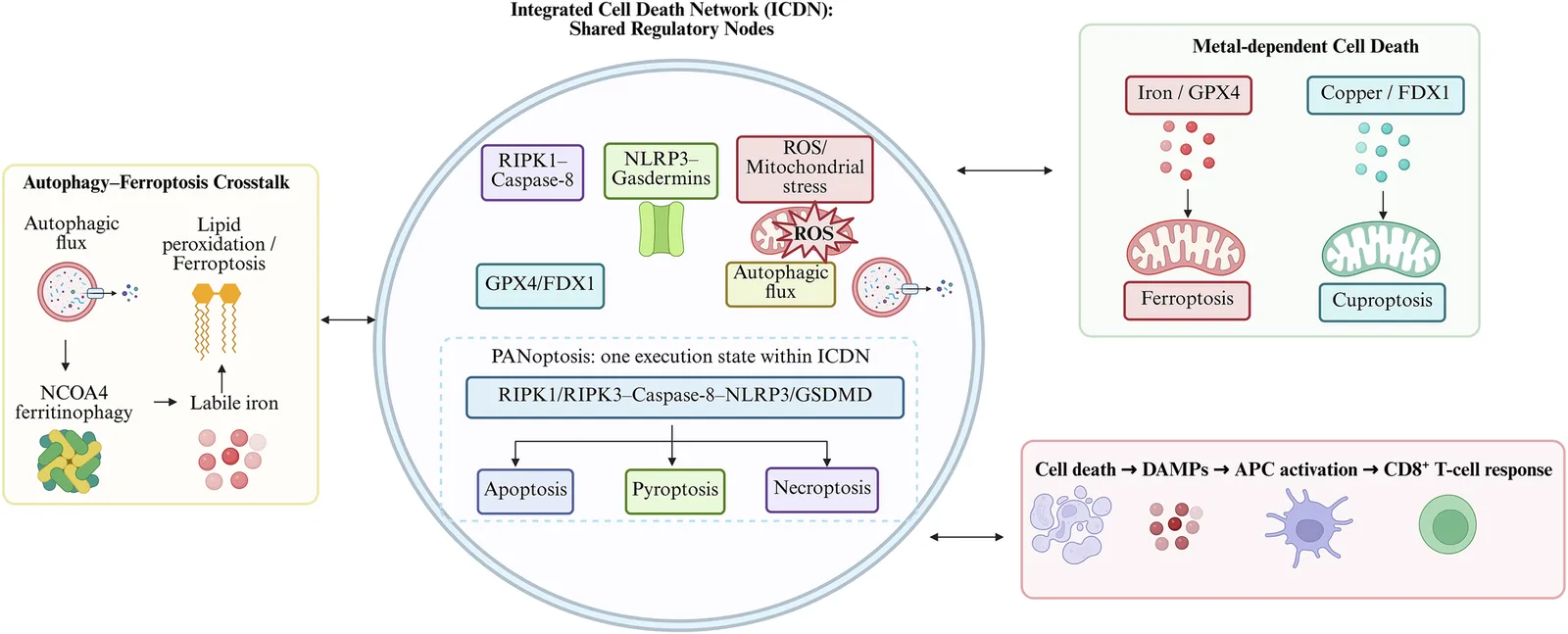
Crosstalk and key integrative nodes within the network of PCD in the inflammatory microenvironment. In the context of inflammation, several PCD pathways are integrated into a densely interconnected regulatory network via shared signaling hubs with other apoptotic and nonapoptotic cell death mechanisms. PANoptosis, driven by the PANoptosome complex, is one execution state of the ICDN and integrates RIPK1, RIPK3, caspase-8 and members of the inflammasome signaling network into a unified signaling platform involving all three cell death modalities. Ferroptosis and cuproptosis are two forms of metal-dependent cell death. Ferroptosis is driven primarily by GPX4 inactivation, which induces lipid peroxidation, whereas cuproptosis is triggered by copper overload, leading to FDX1-dependent aggregation of mitochondrial lipoylated proteins. DAMPs such as HMGB1, ATP and calreticulin (CALR), which promote antigen processing and adaptive immune activation, are released or released during immunogenic cell death. At a larger scale in PCD crosstalk, autophagy, oxidative stress and inflammasome platforms serve as central integrative nodes. These nodes coordinate both synergistic and antagonistic interactions among apoptosis, pyroptosis, necroptosis and ferroptosis via the modulation of ferritinophagy, ROS production and NLRP3 activation. This network governs the spread of inflammatory signaling and ultimately determines the degree of tissue damage

The linkage of several types of PCD into signaling circuits is enabled by inflammatory microenvironments, with these modalities connected by shared regulatory nodes and signaling pathways [11]. Such complex signaling cascades are established in niche sites within dynamically mobilized, inflammation-sculpted tissue shaped by inflammatory stimuli that promote immune cell entry, local release of cytokines and chemokines, accumulation of DAMPs, metabolic and mitochondrial stress, and vascular barrier destabilization [11, 15, 19]. Activated macrophages and recruited neutrophils, in particular, may act as significant amplifiers of inflammatory niches via the sustained release of cytokines and inflammatory mediators as well as cell-to-cell communication [15, 19]. In pathological scenarios, a single cell can engage multiple death programs simultaneously, and among these, control nodes determine which program may take precedence over others [112]. Therefore, PCD should not be conceptualized as parallel independent pathways but instead as a dynamic integrated network. Multiple cell death phenotypes within the same cellular or tissue context may be influenced by common regulatory nodes instead of a single overriding pathway [113]. At the systems level, this crosstalk encompasses several dimensions, ranging from inflammatory signaling platforms, proteolytic systems, and redox status to organelle stress [114]. These factors not only shape inflammatory signaling but also influence the execution of distinct cell death programs [115]. Within the ICDN framework, this section delineates the interconnections among PCD modalities and their roles in inflammation, as shown in Fig. 2.

### PANoptosis

**Fig. 3:**
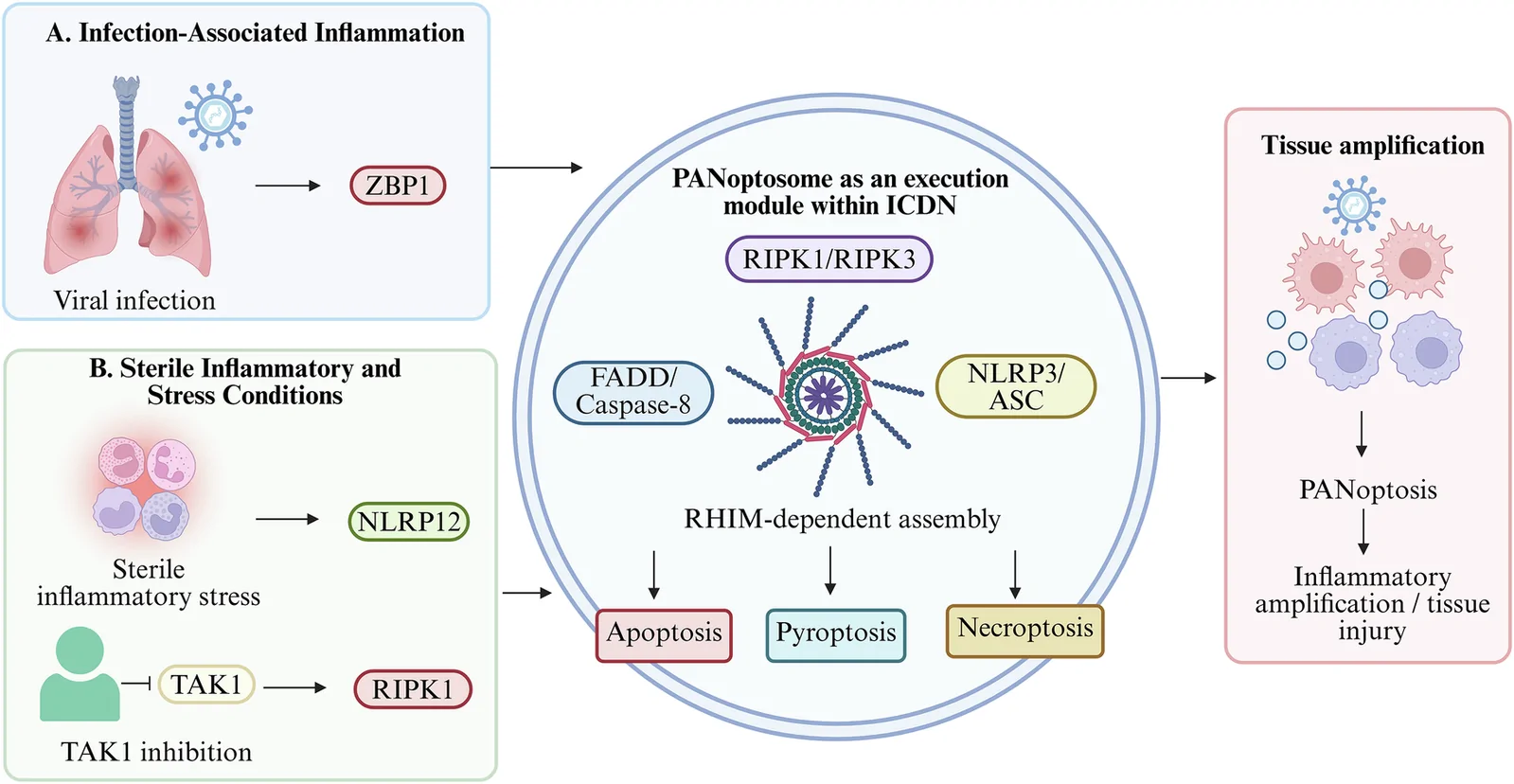
PANoptosome-mediated integration of PANoptotic cell death signaling. In inflammatory microenvironments, various innate immune sensors converge on the PANoptosome to integrate different PCD pathways into a collective signaling network. **A** In the context of viral infections, such as those caused by SARS-CoV-2 or murine hepatitis virus (MHV), the cytosolic sensor ZBP1 senses viral nucleic acids and initiates PANoptosome formation. This complex is structured through RHIM-dependent protein–protein interactions that form a signaling platform that integrates RIPK1, RIPK3, FADD and caspase-8 along with components of the inflammasome, such as NLRP3 and ASC. This integrated platform allows for the simultaneous activation of pyroptosis, apoptosis and necroptosis. **B** In addition to infectious triggers, sterile inflammatory or stress conditions, such as heme in the presence of PAMPs or TNF stimulation facilitate PANoptosome assembly through an NLRP12-dependent axis. Conversely, host sensing of TAK1 blockade can induce RIPK1-dependent PANoptosis

Although multiple effector programs have largely been shown to be synergistically coupled in the context of inflammatory tissues, cell death is not restricted to a single modality. PANoptosis is a collective term that refers to the concurrent activation of apoptosis, pyroptosis and necroptosis in response to the same stimulus, typically through the assembly of a common molecular platform [116, 117]. This is an integrated response that ultimately leads to the lysis of inflammatory cells. Within the ICDN paradigm, PANoptosis is induced by several concurrent death programs that are based on a common signaling scaffold. It is not individual events but the composite realization of multiple trajectories under common conditions, as shown in Fig. 3.

At the molecular level, heterogeneous inflammasome and death-associated proteins aggregate to form a multiprotein complex referred to as the PANoptosome. Importantly, the absence of caspase-1 or GSDMD does not preclude cell death, suggesting the integration of several death pathways in this complex [118]. RIP homotypic interaction motif (RHIM) domain-dependent interactions primarily mediate PANoptosome assembly. Upon sensing by receptors such as ZBP1 or TIR-domain-containing adapter-inducing interferon-β (TRIF), RIPK1/RIPK3 are engaged via the RHIM to construct a signaling scaffold involving both caspase 8 and the NLRP3 apoptosis-associated speck-like protein containing a CARD (ASC) complex, integrating apoptosis, necroptosis, and pyroptosis into the same platform [119]. In infection-associated inflammation, ZBP1 functions as a key cytosolic sensor that mediates β-coronavirus-induced PANoptosis in macrophages and infected lung tissue. Viral infection may also induce similar death responses in neighboring uninfected cells, amplifying tissue-level inflammation. However, extant models based on PANoptosomes focus mainly on the assembly of a particular molecular complex and may not provide complete insight into how these death programs vary across distinct inflammatory contexts [11, 15]. At least in certain pathological contexts, individual death modalities may be regulated by common upstream signals even if the assembly of a classical PANoptosome has not been shown [11, 12]. Moreover, such regulatory switches have not been fully characterized across different cell types, contexts and diseases [12, 15].

Under sterile inflammatory or stress conditions, PANoptosis may also be initiated. An example is heme-coupled pathogen-associated molecular patterns (PAMPs) or TNF, which trigger the NLR family pyrin domain-containing 12 (NLRP12)-dependent formation of proinflammatory inflammasome and PANoptosome complexes, promoting cell death via caspase-8 and RIPK3 [120]. The same applies to RIPK1-dependent PANoptosis driven by loss of transforming growth factor beta-activated kinase 1 (TAK1) function and regulated by the protein phosphatase 6 complex, with IRF1 acting upstream to orchestrate the expression of PANoptosome components [121, 122]. These findings, in turn, imply that PANoptosis is regulated by distinct upstream signals and may have context-selective functions across inflammatory contexts.

Although PANoptosis provides an important mechanistic explanation for the synchronized triggering of apoptosis, pyroptosis and necroptosis, it mainly defines a concerted executional state associated with PANoptosome assembly [123]. On the other hand, the ICDN takes a more synthetic view of regulation by proposing that cell death modalities are not merely pathways but context-dependent outputs controlled by shared nodes in signaling networks. The ICDN is not intended to replace PANoptosis but instead can incorporate PANoptosis as one of many potential execution states within the broader network landscape. This allows ICDN to address additional biological questions, including how cells select one death program over another in different inflammatory environments, the compensatory activation of alternative pathways upon perturbation of one modality, and the spatiotemporal reorganization of death-associated signaling within disease.

While considerable advances have been made in understanding PANoptosome assembly, its cell-type- and disease-specific functions across inflammatory contexts remain incompletely described [124]. During infection, PANoptosis has been shown to support host defense [125] but, under specific conditions, can also aggravate inflammation and disease pathology [120]. Therefore, PANoptosis is best viewed as a context-dependent integrative death modality rather than a uniformly executed pathway [124]. Strategies to selectively modulate PANoptosis across different disease contexts remain incompletely defined [120]; importantly, PANoptosis represents the coordinated execution of multiple death programs through defined PANoptosome complexes [123], whereas ICDN is proposed as a broader regulatory architecture in which multiple PCD modalities interact and are differentially engaged across contexts without necessarily forming a single physical complex, thereby positioning PANoptosis as one experimentally characterized form of coordinated execution within the broader ICDN framework.

### Metal-dependent cell death

Metal-dependent cell death encompasses regulated cell death modes associated with metal ion homeostasis and does not simply represent a mechanistic merger between ferroptosis and cuproptosis. Although connections can be drawn between these two processes with respect to metal homeostasis, oxidative stress, and metabolic regulation, ferroptosis and ferroptosis are mechanistically distinct forms of cell death [99, 126]. Ferroptosis is triggered mainly by iron-dependent lipid peroxidation and the collapse of GPX4-mediated antioxidant defense, whereas cuproptosis originates from copper-induced aggregation of mitochondrial lipoylated proteins and proteotoxic stress [127]. Ferroptosis and cuproptosis are closely related to intracellular metal homeostasis as well as the metabolic state and are therefore considered together here. Mechanistically, however, they are very different. Ferroptosis is caused by iron-dependent lipid peroxidation [128], whereas the causative agent of cuproptosis is the aggregation of lipoylated mitochondrial proteins and proteotoxic stress. These aspects of metal handling capacity, mitochondrial metabolism, and redox state jointly illustrate how they dictate cytotoxicity in the context of inflammation. Recent evidence suggests that fluctuations in metal ion levels may actively influence cell death susceptibility and inflammatory responses [129].

Metal-dependent cell death can potentiate inflammation through metabolic disturbance and damage-associated signaling. In ferroptosis, downregulation of GPX4 promotes lipid peroxidation and is associated with increased malondialdehyde, lipid ROS, and iron levels, thereby facilitating tissue injury [95]. Rab26 is a negative regulator of GPX4 degradation, and Rab26-deficient macrophages are more sensitive to ferroptosis [130]. In contrast, neither deferoxamine (DFO) nor ferrostatin-1 reduces LPS-dependent lung injury, indicating that the process of ferroptosis is not simply a passive consequence of oxidative damage but that it actively participates in inflammatory amplification [96]. A comparable pattern is seen in a cuproptosis-related context. In sepsis-induced cardiomyopathy and other models, disturbed copper homeostasis destabilizes mitochondrial lipoylated proteins, causing tissue injury and inflammation [131]. Mechanistically, both ferroptosis and ferroptosis can promote inflammation under metabolic stress. Nonetheless, whether ferroptosis and cuproptosis converge on common inflammatory outcomes or represent separate context-dependent responses remains unclear. Direct comparative analyses, however, are still few and far between.

Conversely, inflammation changes cellular susceptibility to metal-dependent cell death. Inflammation drives excess ROS, organelle dysfunction, and disrupted metal deposition and transport, leading to altered intracellular metal distribution. In ferroptosis, inflammatory signaling controls GPX4 expression as well as lipid metabolism and iron storage to drive down the threshold for lipid peroxidation [132]. For instance, the transcriptional repression of GPX4 mediated by AP-1 directly connects inflammatory signaling and sensitivity to ferroptosis [133]. In cuproptosis, evidence is still scarce but suggests that oxidative and mitochondrial stress associated with inflammation may modify cytoplasmic copper homeostasis and metabolism, which may alter susceptibility to cuproptotic cell death [134]. Inflammation is therefore seen as more than a background condition but rather an active participant in regulating metabolic and metal-handling processes.

Indeed, the interplay between ferroptosis and cuproptosis is driven mainly by metal homeostasis, redox balance, and mitochondrial functions rather than by shared execution machinery [129]. Ferroptosis is characterized by uncontrolled lipid peroxidation, whereas cuproptosis results from the disruption of lipoylated protein homeostasis. Both, however, are influenced by intracellular metal homeostasis and redox balance [126, 135]. In addition, copper overload affects the expression of ferroptosis-related genes and thus promotes ferroptosis through mitochondrial damage and redox imbalance, providing evidence of regulatory crosstalk rather than the independence of metal-dependent cell death pathways [135]. These observations are consistent with a view within the ICDN framework whereby modalities of death are context-dependent outcomes that arise from shared regulatory conditions.

From a therapeutic angle, focusing on a single pathway may be insufficient to capture the variability across disease models [15]. On the other hand, GPX4 activity, metal ion homeostasis and the mitochondrial metabolic state may represent important determinants of cellular responses to metal-dependent cell death [136, 137]. Inflammation, metal homeostasis, and cell death are therefore not stand-alone processes but are integrated through common regulatory nodes [106]. Future studies linking these pathways to tissue localization and temporal dynamics through experimental validation will be needed to define their contributions to inflammatory diseases.

### ICD and bidirectional communication with the tumor microenvironment (TME)

**Fig. 4:**
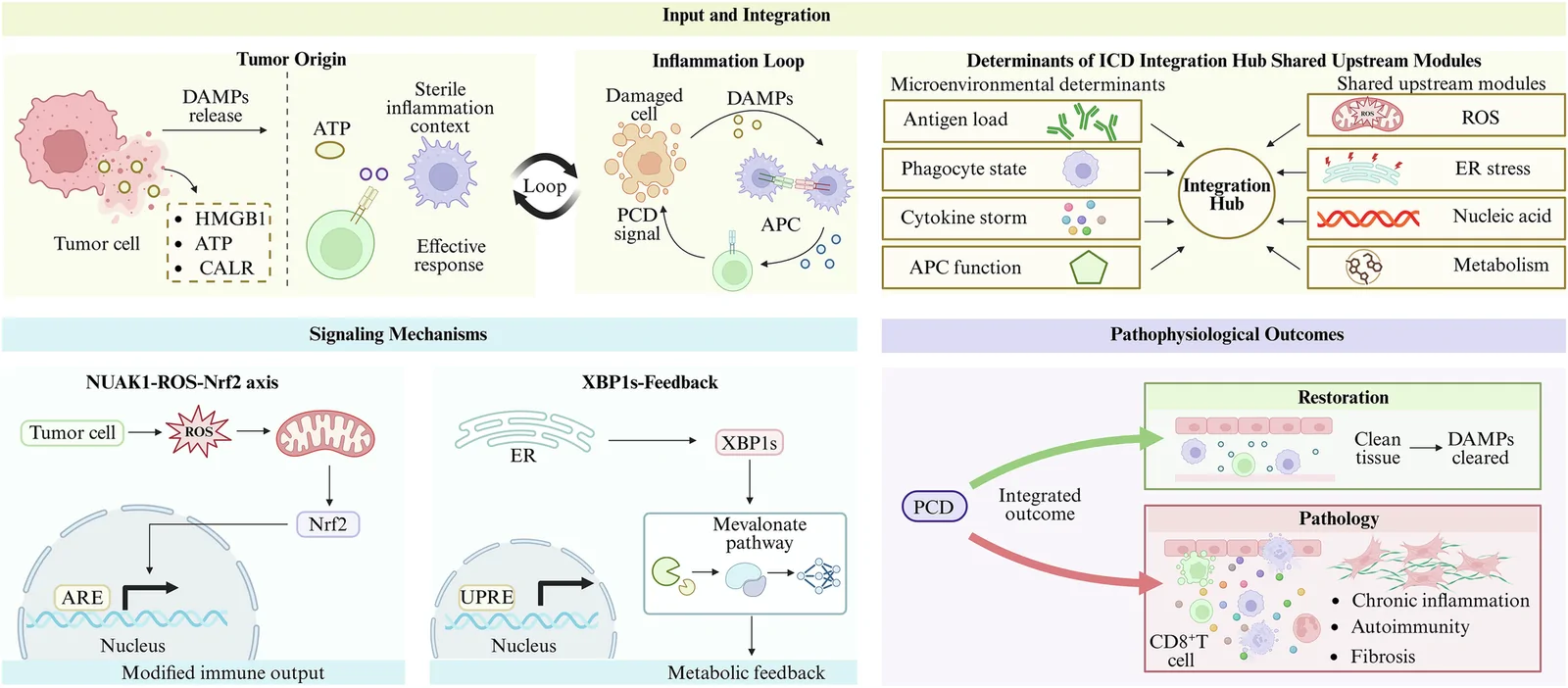
Signal integration in ICD and its impact on the immune microenvironment. ICD is a form of regulated cell death that initiates adaptive immune responses via the release or surface exposure of DAMPs, including HMGB1, ATP and CALR. These signals promote antigen presentation and subsequent immune priming. In the context of tumor or inflammatory microenvironments, DAMPs are sensed by APCs, which activate CD8⁺ T-cell responses and establish a feed-forward inflammatory circuit between cell death and immune activation. The immune readout of the ICD is tightly regulated by microenvironmental conditions, including antigen load, phagocyte status, the cytokine milieu and APC function. ROS generation, ER stress, nucleic acid sensing and metabolic reprogramming act as upstream modules that converge on integrative nodes to coordinate ICD signaling. Specific regulatory axes, including the NUAK1-ROS-NRF2 pathway and XBP1s-driven metabolic feedback, further shape ICD-associated immune outputs. Depending on the integrated microenvironmental context, ICD can either promote tissue repair and DAMP clearance or drive pathological processes such as chronic inflammation, autoimmunity and fibrosis

ICD was initially described as a type of cell death that can induce immunity through the release or exposure of DAMPs during the clearance of cells and increase antigen presentation to activate adaptive immunity [138]. In contrast to PANoptosis, ICD is not restricted to a single molecular pathway; instead, it describes the immunological impact of cell death. In the following, ICD is discussed from the perspective of the immune microenvironment, with a focus on how identical death events manifest different immune outcomes in different contexts, as illustrated in Fig. 4.

ICD alters the local immune environment in the TME. This process relies not only on DAMP release but also on the efficiency with which these signals are sensed by antigen-presenting cells (APCs), resulting in antigen processing, presentation, and T-cell priming [139]. The immunological outcome of ICD is thus also dictated by multicellular cooperative processes ranging from signal emission to immune cell recruitment and downstream activation. In some tumor contexts, APC networks (including macrophages and dendritic cells) are activated by IP, with heightened ICD-associated activity coupled with increased intercellular communication [140]. These findings suggest that ICDs are more than simply dying cells but rather modify the crosstalk of immune cells to dictate local immunity [141]. However, many studies still equate ICD with increased DAMPs, often overlooking whether antigen presentation and functional T-cell responses are effectively established.

In contrast, the TME directly affects both ICD induction and immunological outcomes. How well ICD is converted into potent immunity is intimately linked to oxidative stress, endoplasmic reticulum (ER) stress, the metabolic context and the presence of immunosuppressive cells. Similarly, variations in ROS or responses driven by nuclear factor erythroid 2-related factor 2 (NRF2) modulate immune outputs postdeath, and X-box binding protein 1, spliced form (XBP1s) splicing and metal programs impact the duration of ICD signaling functions [142]. These results highlight that the TME is not a mere passive bystander but an active modulator of ICD signaling. In immunosuppressive terrains, DAMP release may not properly activate immune stimulation, which can lead to disadvantageous antigen presentation and T-cell activation [36, 143]. Notably, systematic comparisons across distinct microenvironmental contexts remain limited.

The ICD, TME, and inflammation constitute a nonlinear, bidirectional regulatory network. Under specific conditions, ICD can improve local immune activation and antigen presentation and mobilize T cells to relieve immunosuppression [144, 145]. However, these same processes can also potentiate proinflammatory signaling, sustain immune cell infiltration, and drive tissue damage. Thus, ICD outcomes are context dependent, ranging from effective immune clearance to enduring inflammatory amplification [141]. Under this framework, ICD is most accurately conceptualized as a bidirectional phenomenon in which cell death remodels the TME, which in turn prescribes whether such a T-cell-mediated response is immunogenic. This context dependency clearly differentiates ICDs from PANoptosis, the latter underlining that coordinated terminal execution of multiple death pathways occurs under common stimuli, whereas ICDs underscore the environment-dependent transformation of cell death into immune outcomes.

From a therapeutic perspective, the net benefit of ICD-based countermeasures depends not only on maximizing cell lysis but also on increasing immunological detection and uptake. The overall antitumor efficacy is dictated by DAMP release, antigen presentation capacity, T-cell recruitment and the immunosuppressive status of the TME [146]. Manipulation of ROS, ER stress and metabolic or epigenetic states can alter the ICD induction process and its downstream immune effects, potentially altering therapeutic responses [146–148]. As highlighted in Table 2, heterogeneous ICD inducers orchestrate metabolic reprogramming and upregulate inflammatory signaling and immune cell function, cumulatively leading to TME remodeling and enhanced responses to immunotherapy across multiple tumor models.

**Table 2:** Key mechanisms, intervention strategies, and application contexts for inflammation-driven remodeling of the tumor immune microenvironment

PCD/ICD-related remodeling strategies	PCD/ICD-related regulatory nodes	Representative Experimental Readouts (Common Metrics)	Key Interactions with the TME	Major Disease/Model Contexts	Potential Therapeutic Implications	References
CBD-mediated immune microenvironment remodeling associated with macrophage functional reprogramming	PI3K–AKT signaling inhibition and macrophage functional reprogramming	Immune-state profiling by Single-cell RNA sequencing (scRNA-seq)/scATAC-seq	Suppression of M2 phenotype and enhancement of M1 antitumour activity	MC38 murine colon carcinoma xenografts and human CRC organoid coculture models	Enhanced responsiveness and sensitivity to PD-1 immunotherapy	[240]
IL1R2 blockade-mediated inflammatory immune remodeling	IL-1 inflammatory signaling regulation and immune suppression reversal	Assessment of tumor-associated macrophage (TAM) polarization and CD8⁺ T-cell exhaustion	Reduced TAM recruitment and alleviation of immunosuppression	Triple-negative breast cancer mouse models	Improved efficacy of PD-1 blockade	[241]
Annexin A1 (ANXA1) deficiency-driven accumulation of apoptotic cells	ANXA1-associated efferocytosis regulation and cGAS/STING immune activation	c-Caspase-3 expression and macrophage profiling	Impaired apoptotic clearance and activation of pro-inflammatory immunity	Pancreatic cancer patient samples and mouse models	Enhanced efficacy of chemotherapy combined with PD-1 blockade	[242]
Inhibition of tumor glycolysis combined with immune blockade	GLUT1–lactate–IFNγ–PD-L1 regulatory axis	TAM/Treg ratio and effector T-cell activity	Relief of metabolically immunosuppressive microenvironment	Orthotopic glioblastoma mouse models	Enhanced efficacy of vaccination and PD-1 blockade	[243]
Inflammation-driven bone marrow microenvironment reprogramming	1q gain subclones and upregulated inflammatory factors	Clonal heterogeneity resolved by single-cell transcriptomics	Enhanced immunosuppressive myeloid interactions	Relapsed/refractory multiple myeloma	Guidance for resistance stratification and therapeutic decision-making	[244]
Lenvatinib-associated immune microenvironment modulation	Immune checkpoint regulation and inflammatory microenvironment remodeling	Flow cytometry integrated with transcriptomic immune profiling	Reduction of Tregs and activation of the immune microenvironment	Syngeneic hepatocellular carcinoma mouse models	Identification of populations benefiting from combination therapy	[245]
Plant-derived exosome-induced oxidative stress	ROS elevation, apoptotic signaling and M1 polarization	ROS levels and macrophage phenotyping	Promotion of pro-inflammatory immunity and TME remodeling	4T1 triple-negative breast cancer model	A low-toxicity, natural nanotherapeutic strategy	[246]
Androgen deprivation therapy-induced immune remodeling	AR–PD-L1 upregulation and suppression of inflammatory signaling	Transcriptomic analysis and immune cell flow cytometry	Promotion of myeloid-derived suppressor cell (MDSC) accumulation and M2 polarization	Castration-resistant prostate cancer models	Mechanistic insight into failure of PD-L1-based combination therapy	[247]
Caspase-1-dependent pyroptotic signaling-associated immune microenvironment remodeling	Caspase-1, pyroptotic signaling and inflammatory cytokine release	CAF subtype markers and collagen expression analysis	Inflammatory CAF–CSC feedforward loop driving resistance	Chemoresistant bladder cancer patients and models	Indicates the dual-edged role of inflammatory cell death	[248]
Triggering receptor expressed on myeloid cells 2 (TREM2) knockdown induces pro-inflammatory immune activation	TREM2-associated myeloid immune regulatory signaling	Assessment of ROS, DNA damage and apoptosis	Pro-inflammatory microglia-mediated tumor suppression	Orthotopic glioma mouse models	TREM2 as a novel immunotherapeutic target	[249]
Combined mitogen-activated protein kinase kinase (MEK) and STAT3 inhibition	MEK/STAT3 signaling and stromal immune regulatory nodes	scRNA-seq, cytometry by time-of-flight (CyTOF) and immune profiling	CAF plasticity drives immune reprogramming	KRAS-driven orthotopic pancreatic ductal adenocarcinoma (PDAC) models	Overcoming immunotherapy resistance in PDAC	[250]
PANoptosis-associated immune microenvironment activation	ZBP1/RIPK1/RIPK3/caspase/gasdermin-associated PANoptotic signaling	ROS levels, DC maturation and CTL activation	Tumor-specific inflammatory amplification of immune responses	Orthotopic osteosarcoma and bone defect models	Precision sonodynamic immunotherapy with bone repair	[251]
HDAC6 inhibition induces inflammatory ICD	HDAC6-associated inflammatory signaling and immune activation	CD8⁺ T-cell infiltration and macrophage polarization	Remodeling toward a pro-inflammatory, immunologically active microenvironment	Melanoma and colon cancer models	Enhanced responsiveness to PD-1 inhibitors	[252]
Therapies targeting DNA repair deficiency	Homologous recombination repair deficiency and PD-L1 expression	cfDNA sequencing and tumor-infiltrating lymphocyte immune phenotyping	Enhanced immune infiltration and checkpoint activation	Inflammatory breast cancer patient samples	Guidance for poly(ADP-ribose) polymerase (PARP) inhibitor and immunotherapy combinations	[253]
Anthracycline-induced ICD-associated immune microenvironment activation	CALR exposure, ATP release and HMGB1-associated ICD signals	Dendritic cell activation and T-cell expansion	M2-to-M1 repolarization and relief of immunosuppression	In situ tumor vaccination mouse models	Amplification of vaccine effects and enhancement of immunotherapy	[254]
TME-associated NK-cell functional suppression	Immune checkpoint receptor-associated NK cell regulatory nodes	Reduced perforin/granzyme levels and decreased IFN-γ	TME-driven NK cell exhaustion and functional reprogramming	Tumor tissues from patients with non-small cell lung cancer	Indicates therapeutic potential of targeting NK cell checkpoints	[255]
Irreversible electroporation-induced inflammatory cell death	Inflammatory cell death-associated immune activation signals	G0/G1 arrest and enhanced IFN-γ signaling	Transient immune activation followed by suppression	Orthotopic pancreatic cancer mouse models	Suggests the need for combination with immune checkpoint blockade	[256]
Epidermal growth factor receptor (EGFR) inhibitor-induced inflammatory responses	Reduced phosphorylated EGFR/ERK and upregulation of inflammatory genes	Increased CD3⁺/CD8⁺ T-cell infiltration	Immune activation accompanied by CAF/EMT remodeling	Window-of-opportunity study in primary HNSCC	Expands the population likely to benefit from PD-1 blockade	[257]
Inflammation-associated PD-L1 upregulation and immune checkpoint regulation	PD-L1-associated inflammatory immune regulatory signals	Immunohistochemistry assessment of PD-L1 and CD3/CD68	PD-L1-associated heterogeneity in immune infiltration	Gastrointestinal stromal tumor patient cohorts	Provides a basis for immunotherapy stratification	[258]
Antibody‒drug conjugate and bispecific antibody-associated ICD activation	Tumor antigen release and immune activation-associated ICD signals	Enhanced CD8⁺ infiltration and dendritic cell maturation	Conversion of “cold” tumors into inflamed “hot” tumors	Triple-negative breast cancer and metastatic models	Improved overall efficacy of immunotherapy	[259]

Despite these advances, several limitations remain. Elevated ICD markers are often interpreted as a sign of functional immunity, without any verification of downstream antigen presentation or T-cell activity. Inconsistent definitions of ICD further limit comparability between studies. In addition, whether ICD enhances protective immunity or pathogenic inflammation is context dependent [149]. Future investigations need to incorporate cell death signaling pathways, microenvironmental temporal and spatial dynamics, and in vivo inflammatory endpoints within single conceptual frameworks that can provide more precise functional descriptions of ICDs across disease contexts.

### Cross-regulation of other PCD modalities (synergistic/antagonistic interactions among autophagy, apoptosis, ferroptosis, pyroptosis, and necroptosis)

Inflammatory injury rarely involves isolated forms of PCD but rather their coordination by shared regulatory nodes such as autophagy, oxidative stress and inflammasome platforms. These nodes are not strictly pathway specific but rather modulate initiation thresholds and execution strength across multiple death modalities. Persistent ROS accumulation, for example, increases cellular sensitivity to lipid peroxidation, a process that can lead to ferroptosis [150], as well as destabilize lysosomes (which activate inflammasomes and pyroptosis) [151]. Such observations support a model of divergence from shared hubs rather than independent or additive pathways.

Autophagy acts as a master integrator in this network. Moreover, nuclear receptor coactivator 4 (NCOA4)-mediated ferritinophagy during ferroptosis increases the level of intracellular labile iron and drives lipid peroxidation [152]. Mitochondrial ROS further promote autophagy through the AMP-activated protein kinase (AMPK)-unc-51-like autophagy-activating kinase 1 (ULK1) pathway in zinc oxide nanoparticle (ZnONP)-induced ferroptosis, enhancing the ferroptotic response [153]. Processes associated with autophagy also alter ferroptosis thresholds during ischemia–reperfusion, where mTORC1-dependent control of OTU domain-containing deubiquitinase 5 (OTUD5) stability regulates GPX4 levels and increases sensitivity to ferroptosis [154]. These results reveal that autophagy mediates damage clearance and regulates iron metabolism and antioxidant capacity, ultimately shaping ferroptotic susceptibility.

Context-dependent switching between pyroptotic and apoptotic outputs has also been reported in experimental systems. In renal tubular epithelial cells treated with chemotherapeutic agents, activation of caspase-3 cleaves GSDME to cause pyroptosis-like cell death; inhibition of GSDME in the same context skews the resulting stimuli toward apoptosis, as evidenced by an increase in cell viability and decreased lactate dehydrogenase (LDH) release [155]. Thus, homologous upstream signals could be directed to different death programs according to the intracellular levels of pivotal executioners. Moreover, in addition to its canonical significance in necroptosis, RIPK3 modulates autophagic flux, and its interaction with the p62-Keap1 axis suggests that there is bidirectional crosstalk between necroptosis and autophagy [156].

Some signaling nodes are capable of simultaneously linking three or more PCD modalities. Such schemes are well established, such as those involving the NLRP3 inflammasome and mitochondrial ROS. In ischemia–reperfusion models, NLRP3 blockade suppresses neuronal ferroptosis, while mitochondria-derived ROS mediate apoptosis and necroptosis but also induce pyroptosis by inducing lysosomal membrane permeabilization and the release of active cathepsin B, leading to the activation of NLRP3 [157, 158]. While direct interconversion among these death states is still inadequately demonstrated, ferroptosis, apoptosis, necroptosis and pyroptosis may coexist or share upstream regulatory inputs in this mitochondrial ROS–lysosome–NLRP3 axis.

Pathologically, node-centric crosstalk is a hallmark of the amplitude and duration of inflammation. Sustained activation of shared nodes can initiate many death mechanisms, fueling tissue damage [157]. On the other hand, inhibition of these nodes can have multiple pathway therapeutic effects. ROS are a prime example of such a hub, as high levels of ROS induce ferroptosis via lipid peroxidation, induce apoptosis through mitochondrial damage and increase pyroptosis via NLRP3 inflammasome activation, thus coordinating multiple death programs in a single inflammatory context [159, 160]. This framework helps rationalize why single-pathway interventions generally have low efficacy but therapeutic approaches aimed at common regulatory nodes may better capture the multimodality of disease.

Nevertheless, current studies remain fragmented. Most available studies focus on specific models or individual regulatory nodes, with relatively few systematically comparing multiple PCD modalities within unified experimental systems. Temporal ordering and causal relationships among death pathways are poorly defined, and existing evidence is largely correlative. Future studies should develop strategies that combine dynamic monitoring, spatial resolution and multipathway perturbations to better understand the functional roles of shared nodes in inflammatory diseases. Establishing a network-level framework is critical for defining the structural and therapeutic features of ICDNs under varying inflammatory conditions.

## Targeted therapeutic strategies

PCD should not be interpreted as a multiple-independent therapeutic target in the context of inflammation-associated diseases. Instead, inflammatory environments reshape the PCD network by altering upstream signals and regulatory nodes to modulate disease-specific execution biases. Therapeutic approaches should not be aimed at blocking death pathways by themselves outside the context of the disease, its inflammatory stage, or the underlying network configuration. Cell death can be manifested through multiple modalities that may coexist or occur in series during the same disease course, and inhibition of a single pathway can result in compensatory activation of an alternative pathway [10, 161, 162]. In this sense, the ICDN framework highlights that disease-specific network states need to be characterized, along with therapeutic windows where immune regulation could be balanced with tissue protection. Importantly, the inflammatory microenvironment proposed by the ICDN is not a disease-specific phenomenon but may arise in relevant regulatory contexts across a large spectrum of pathologies ranging from chronic infection, autoimmunity, and sterile inflammation to tissue injury.

**Fig. 5:**
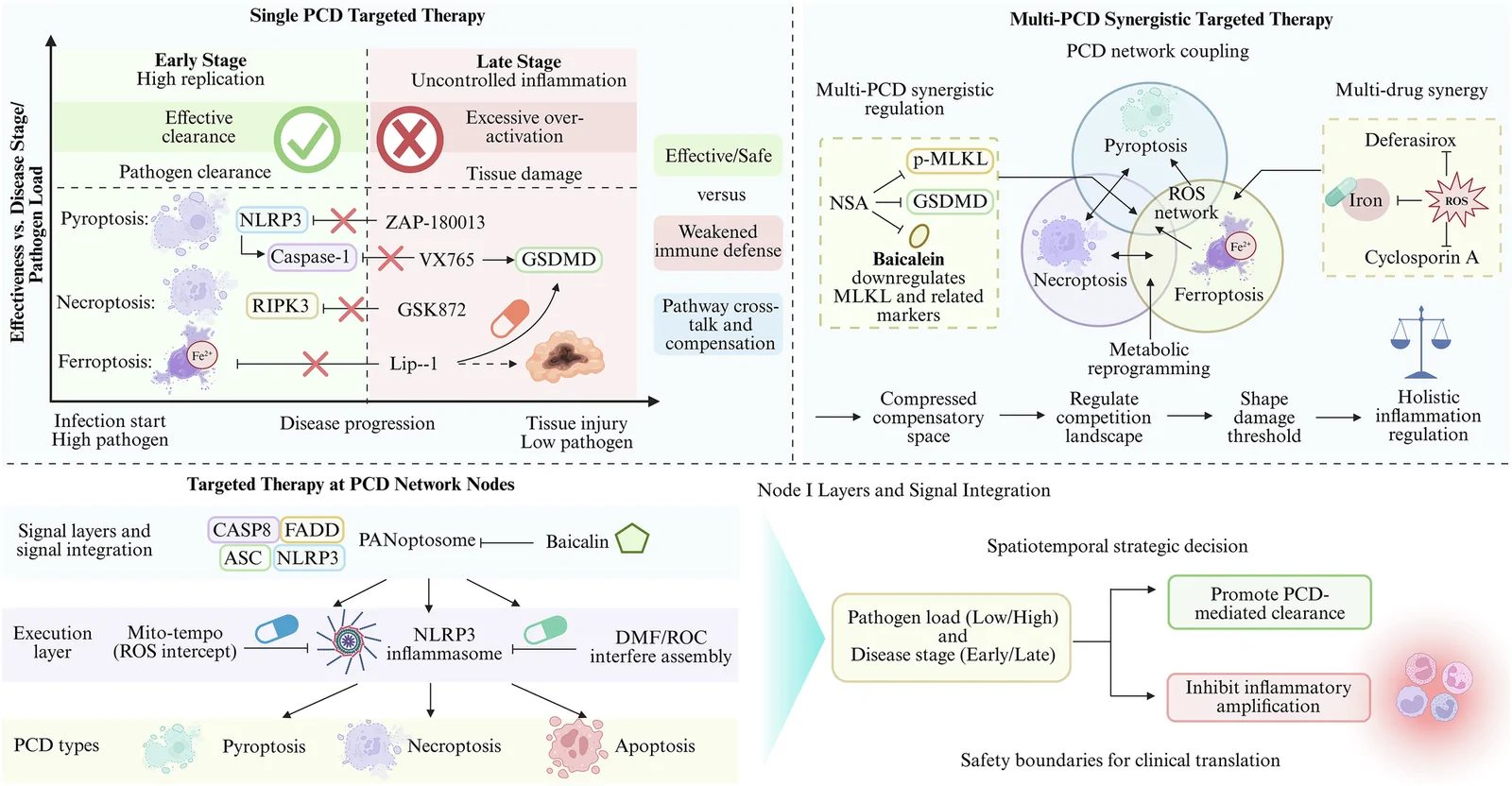
Targeted intervention strategies within PCD networks and their regulatory architecture in inflammation. Distinct forms of PCD, embedded in a highly intertwined network with pervasive signaling crosstalk and functional complementarity, may be coopted during inflammation-associated diseases. Therapeutic strategies targeting this network can be classified into three categories: selective inhibition of individual PCD pathways, combination regimens against multiple PCD pathways, and integrative therapies targeting central regulatory nodes within the PCD network. Selective targeting usually involves the inhibition of certain PCD pathways (pyroptosis, necroptosis or ferroptosis) to reduce inflammatory tissue damage. However, these approaches may come at the cost of host immune competence during disease progression. In contrast, the combinatorial modulation of multiple PCD pathways seeks to prevent compensatory activation between distinct death programs and thus augment global control over inflammatory responses. A different approach focuses on major signaling nodes in the PCD network, such as the inflammasome or PANoptosome, which are able to integrate convergent signals from upstream transduction pathways and coordinate multiple cell death outputs. By targeting these nodes, higher-order regulation across convergent pathways can be achieved. This latter point is particularly important since therapeutic design must account for the delicate interplay between pathogen burden and disease stage to find an optimal balance between achieving pathogen clearance and avoiding excessive amplification of inflammation. Such context-dependent modulation is critically important for maximizing the safety and benefit of interventions in inflammatory diseases

On the basis of this network perspective, the development of innovative therapies to modulate PCD can be classified into three key strategies, namely, selective manipulation of distinct death pathways; simultaneous targeting and coregulation of multiple distinct PCD modalities with a focus on preserving organismal homeostasis; and receptor- or signaling node-centered activation targeting strategic regulatory nodes within a dynamic, interconnected PCD signaling network. These strategies and their regulatory architecture are summarized in Fig. 5. These strategies span from single-pathway interventions to translational studies. The therapeutic efficacy depends on the selection of a molecular target together with the timing of treatment, disease stage and preservation of key host defense functions [163]. Compared with multiple PCD-associated targets that have progressed in the clinic [164, 165], most ferroptosis- and cuproptosis-based interventions are still under preclinical development. More precise definitions to inform disease-stage specificity in therapeutic designs will be necessary for future work to maximize treatment efficacy while limiting tissue damage due to dysregulated immunity.

### Single-PCD targeted therapy

**Fig. 6:**
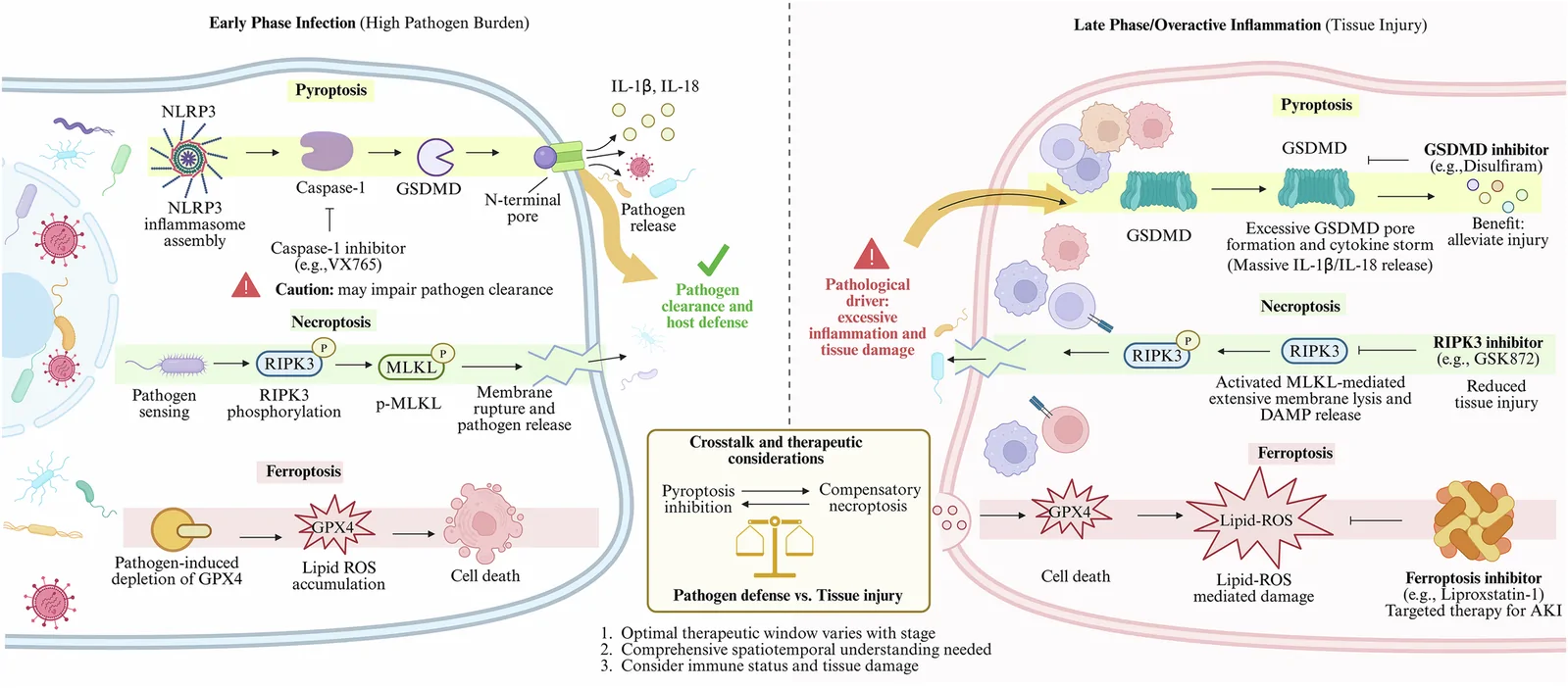
Stage-specific effects and therapeutic windows for targeting individual PCD pathways during infection. Diverse PCD modalities (pyroptosis, necroptosis and ferroptosis) orchestrate pathogen clearance and reinforce host defense during the early stage of infection. For instance, caspase-1 activation by the NLRP3 inflammasome followed by GSDMD pore formation facilitates the secretion of IL-1β and IL-18. Concurrently, RIPK3-MLKL-dependent necroptosis facilitates pathogen release and immune activation. In contrast, in settings of dysregulated inflammation or late in the course of infection, excessive activation of these death programs can lead to excessive release of inflammatory mediators that worsen tissue damage. Thus, specific inhibition of single PCD pathways, for example, by targeting GSDMD or RIPK3 and inhibiting ferroptosis, may provide therapeutic benefit through the inhibition of late-stage inflammation. However, owing to extensive crosstalk between PCD pathways, inhibition of a single pathway may initiate compensatory activation of alternative cell death mechanisms. Clinical implementation, therefore, needs to be carefully aligned with pathogen burden, disease stage and tissue damage to determine an appropriate therapeutic window

This is significant, as inhibition of a single PCD pathway is currently the most clinically tractable approach because of the well-defined molecular targets and often simple drug design and evaluation of potent drug pharmacodynamic responses. However, the best current data suggest that the major obstacle is not a lack of actionable targets but how pathway dependence varies with the stage of disease. Inflammation-related disorders, for example, could involve one such PCD pathway that plays different pro- or anti-inflammatory roles during the initial phase of infection, during the amplification phase of the inflammatory response and during tissue injury. Such functional plasticity as a function of stage indicates that single-pathway approaches might have their genesis in effective preclinical models but ultimately lack clinical validity because of the potential for heterogeneous therapeutic responses [119]. Indeed, individual PCD pathways may play divergent roles between early infection and uncontrolled inflammation, rendering such approaches inherently stage dependent; the principal regulatory mechanisms and potential therapeutic windows are summarized in Fig. 6.

As such, for pyroptosis-targeted therapies, efforts have moved beyond the inhibition of downstream executioners (e.g., caspase-1) and instead focus on modulating the upstream NLRP3 inflammasome to clamp down inflammation and cell death at a much broader scale. The NLRP3 inflammasome, which is upstream of IL-1β release and amplifies inflammation, acts as a central regulatory hub in chronic inflammatory diseases and has thus emerged as an important drug development target in recent years [164]. Structure-guided design directed at its functional moieties and regulatory interfaces is progressing well, with multiple candidate molecules already in clinical development. In contrast, other agents, such as VX-765 and disulfiram, have mainly been used as validation tools for the underlying mechanisms, but their clinical efficacy is as yet untested.

Therapeutic strategies that focus on necroptosis have advanced more swiftly to the clinic. RIPK1, a key molecular node between inflammatory signaling and PCD, has emerged as an attractive drug target. Expanding the mechanistic understanding of the RIPK pathway has enabled the development of inhibitors, some of which are currently being explored in neuroinflammatory and systemic inflammatory diseases [166]. These agents may be effective in select patient populations; however, the cell type-specific functions of RIPK1 are likely to impact the therapeutic response [167]. Thus, future progress in this area will be predicated more on improved approaches to stratifying patients than by the mere inhibition of the target itself.

In comparison, ferroptosis-based treatments are still mostly limited to preclinical applications. These iron chelators and lipid peroxidation inhibitors have been shown to be protective in several models of tissue injury, but their efficacy is context dependent across disease states. New insights have shown that ferroptosis is highly integrated with metabolic mechanisms, immune microenvironments, and other cell death mechanisms, thus leading to substantial context dependence when this pathway is sought in isolation [168]. On the basis of the current data, ferroptosis should likely be considered a potential paradigm for therapeutic exploration at this time instead of a pathway suitable for a stable clinical context.

Unsurprisingly, single PCD-targeted strategies enjoy a comparably clear developmental framework and are amenable to standardized drug development pipelines. However, their effectiveness is often tuned by disease progression and crosstalk among cell death pathways. Previous research suggests that targeting a single pathway can provide measurable benefits in specific instances; however, its efficacy still appears to be limited in heterogeneous inflammatory environments. Taken together, these results demonstrate that single PCD-targeted strategies are best poised as early therapeutic interventions and highlight the potential for synergy in multipathway or nodal regulatory approaches to achieve sustained clinical efficacy. In the context of ICDN, this limited efficacy of single-pathway blockade could represent the adaptive redistribution of signaling flux toward alternative death programs rather than mere failure of target engagement.

### MultipCD synergistic targeted therapy

**Fig. 7:**
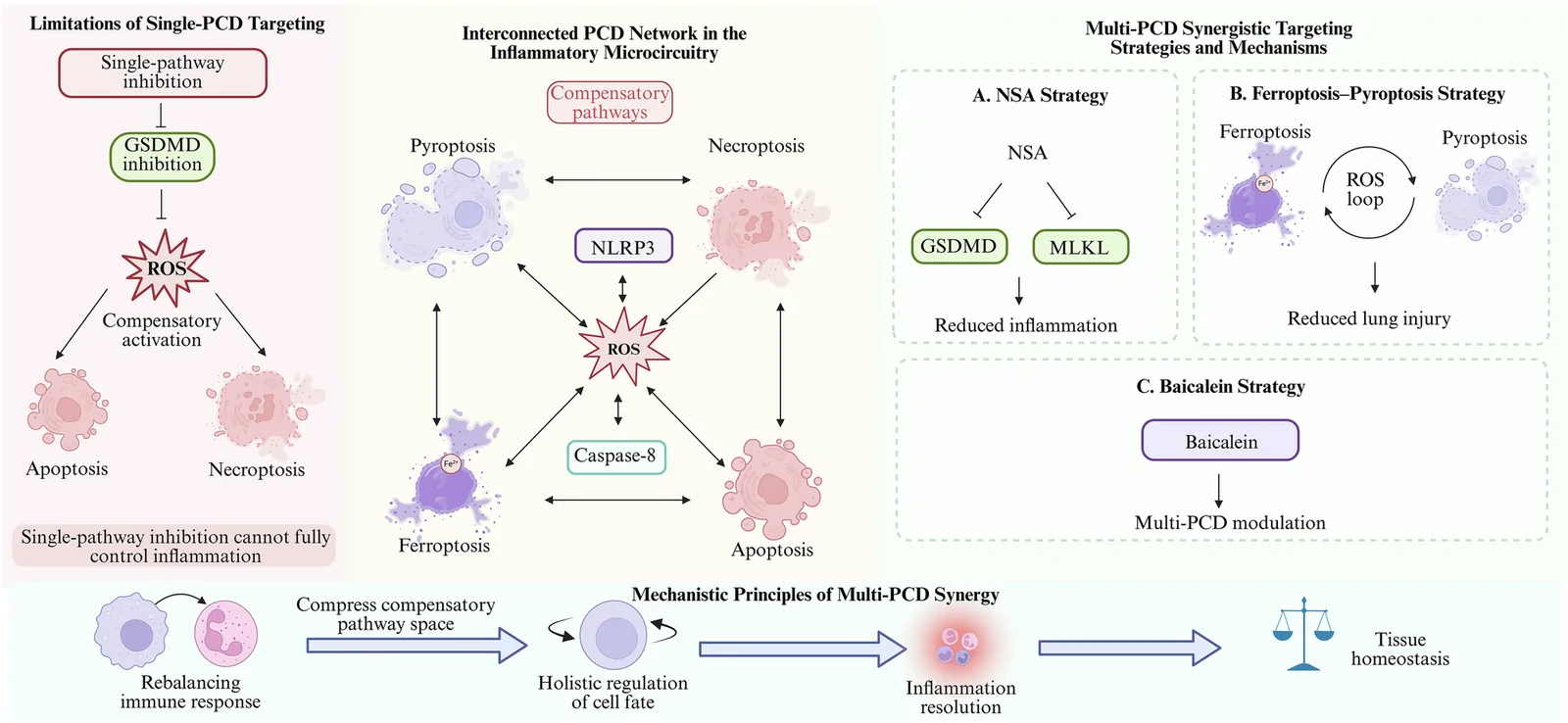
Network regulation of multiple PCD pathways and coordinated targeting strategies in the inflammatory microenvironment. The left panel depicts the problems associated with single PCD pathway targeting. While direct inhibition of individual effectors such as GSDMD can block pyroptotic signaling, numerous cell death processes converge on common stress nodes, especially ROS, and this convergence can lead to compensatory engagement of alternative pathways such as necroptosis and apoptosis. Such redundancy limits the effective regulation of inflammation. The central panel depicts the PCD network architecture shaped by background signals in the inflammatory microenvironment. Pyroptosis, necroptosis, ferroptosis and apoptosis are linked by common signaling hubs, including the NLRP3 inflammasome, caspase-8 and ROS. Thus, they create a tightly integrated regulatory network. The right panel outlines multitargeting strategies involving multiple types of PCD and their underlying mechanisms. NSA simultaneously represses GSDMD and MLKL expression to reduce inflammation. In septic lung injury, a reactive oxygen species (ROS) loop that amplifies ferroptosis and pyroptotic signaling is established; dual inhibition breaks this loop, alleviating tissue damage. Pleiotropic modulators such as baicalein can simultaneously modulate multiple cell death pathways. These combined approaches compress the compensatory landscape at the network level by simultaneously targeting several PCD pathways and thus reducing inflammation and promoting tissue homeostasis more efficiently

MultipCD synergistic targeting involves the simultaneous modulation of multiple PCD pathways by the use of combination therapies or broad-spectrum interventions to blunt compensatory responses secondary to the inhibition of a single pathway. This idea is mostly based on clinical observations. In the context of complex inflammatory diseases, targeted therapies that target a single pathogenetic pathway have insufficient long-term efficacy [169], which in some cases results in relapsing inflammation or progressive organ dysfunction during treatment after initial improvement is observed. As a result, the strategic frameworks, key regulatory mechanisms and potential therapeutic approaches for multi-PCD targeting are summarized in Fig. 7. In clinical practice, however, these approaches remain mainly empirical and are not guided by a systems-level understanding of cell death networks. Classical protocols to treat infections, autoimmune disease and acute organ injury are typically based on anti-inflammatory drugs combined with immunosuppressants and antioxidant therapies [170–172]. Although these interventions can dampen inflammation and ameliorate end-organ performance in some patients, the overall efficacy remains variable, and long-term outcomes are inconsistent.

Different pathways of cell death exhibit considerable variability in their clinical tractability. For example, necrosulfonamide (NSA) has been reported to simultaneously block phosphorylated MLKL and GSDMD-related signaling in animal models, which reduces inflammation in dextran sulfate sodium (DSS)-induced colitis [173]. While these approaches can abrogate inflammatory amplification in vitro, their effects on long-term host defense against infection are unknown. Under oxidative stress, ferroptosis and pyroptosis can establish a positive feedback loop; pharmacologic combined suppression of these processes ameliorates tissue injury in models of septic pulmonary injury [174, 175]. However, given the dynamic immune states across different stages of sepsis, such strategies likely require precise temporal optimization. Baicalein has been reported to downregulate the expression of multiple cell death markers in a dose-dependent manner in vivo [176], suggesting a broad network-modulatory effect, although its molecular specificity and long-term safety require validation in large-scale clinical trials.

Theoretically, cotargeting PCD pathways is a more accurate representation of the complexity of the inflammatory microenvironment. Nonetheless, significant translational challenges remain. Different mechanisms of cell death differentially affect multiple stages of disease pathogenesis; therefore, combining treatments may not only inhibit pathogenic mechanisms but also elicit more nuanced immune responses that increase the likelihood of adverse effects and drug‒drug interactions. Currently, the lack of patient stratification and clearly defined therapeutic windows hinders clinical application, but multiple pathway targeting is still in an exploratory state.

### Targeted therapy at PCD network nodes

Unlike the aforementioned multipathway intervention, modulating key nodes within the PCD network is anticipated to affect a small number of upstream or integrative regulators that dictate multiple forms of cell death for system-level control over inflammatory responses. This approach is based on in vitro and in vivo data supporting a common action of certain hub molecules across cell death pathways as well as more complex global regulatory effects beyond solely pathway modulation.

At the clinical level, node-targeted strategies are being investigated and are entering initial applications. Among these classes, inhibitors of the NLRP3 inflammasome are among the most developed. A number of small-molecule NLRP3 inhibitors have advanced into clinical trials for diseases, including metabolic disorders, autoimmune disorders and neuroinflammation. An example is DFV890, which showed favorable tolerability and clinical improvement trends in a phase II trial involving COVID-19-associated pneumonia [177]. The clinical development of some NLRP3 inhibitors (e.g., MCC950) has ceased because of hepatotoxicity, nevertheless illustrating persistent safety constraints [178].

In addition to targeting inflammasomes, RIPK1 has been identified as a major regulatory node that integrates inflammatory signaling with effector cell death pathways, such as apoptosis and necroptosis. RIPK1 inhibitors, as a new class of small-molecule agent, have shown favorable safety, tolerability and pharmacokinetic profiles in phase I trials and may be useful for both inflammatory diseases and neurodegenerative disorders [179]. Their therapeutic benefit derives from the simultaneous modulation of inflammatory signaling and necroptosis, with efficacy shown in a wide range of disease models, although their specificity needs more systematic testing [180]. In addition, agents that modulate ROS, mitochondrial function or transcriptional regulators are widely used in clinical settings for inflammation control. Although these interventions do not directly target specific cell death pathways, they frequently influence multiple processes, including apoptosis, pyroptosis, necroptosis and ferroptosis [181], and can thus be considered indirect regulators of PCD network nodes.

With respect to the ICDN, single-pathway inhibition cannot fully explain this coordination between distinct cell death modalities. In some cases, targeting critical regulatory nodes in cell death pathways might simultaneously modulate several types of cell death. However, these nodes often have dual functions in immune defense and tissue homeostasis; hence, prolonged inhibition may lead to immune imbalance and unexpected risks [182]. In addition, the dependence on specific signaling nodes is disease model dependent, and the effects of such interventions on immune-mediated cytotoxicity demonstrate considerable heterogeneity [183]. The evidence to date is predominantly based on in vitro studies and limited in vivo validation; thus, long-term outcomes are still lacking. Consequently, future investigations must incorporate molecular profiling with biomarker discovery and patient stratification to characterize target populations and refine therapeutic approaches. Representative PCD-targeted therapeutic strategies and their supporting evidence in inflammation-associated diseases are summarized in Table 3.

**Table 3:** Regulatory strategies targeting PCD and supporting evidence in inflammation-associated diseases

Targeted PCD modality or network axis	Core therapeutic objective (inflammatory context)	Key molecular targets	Representative interventions (commonly used in experimental settings)	Representative disease models	Evidence status and translational considerations	References
RIPK3-mediated necroptosis	Suppress virus-induced lethal inflammation	RIPK3–MLKL signaling axis	RIPK3 inhibitors or MLKL knockout	Severe fever with thrombocytopenia syndrome virus hemorrhagic fever virus infection model	RIPK3–MLKL necroptosis is linked to SFTSV lethality; clinical targeting remains unproven	[50]
ER stress–mediated apoptosis and pyroptosis	Alleviate metabolic inflammation and protect β cells	BI-1–inositol-requiring enzyme 1 alpha (IRE1α)–NLRP3 axis	IRE1α RNase inhibitor STF	Obesity-associated type 2 diabetes model	IRE1α inhibition improves β-cell stress and metabolic injury in models	[260]
STING-associated pyroptosis and necroptosis imbalance in dendritic cells	Attenuate autoimmune inflammatory cell death	STING-CASP1/GSDMD and RIPK3/MLKL axes	STING shRNA knockdown in RAW264.7 validation experiments	Systemic lupus erythematosus and lupus nephritis	Omics and knockdown data support STING-linked pyroptosis/necroptosis	[261]
PD-1/PD-L1-linked immune tolerance regulation	Restore intestinal immune tolerance and suppress colitis-associated inflammation	PD-1/PD-L1-Foxp3/Treg axis	Bifidobacterium infantis administration; anti-PD-L1 blockade used for pathway validation	DSS-induced murine colitis model	DSS mouse data support B. infantis-linked PD-1/PD-L1-Treg regulation	[262]
Inflammatory PCD in microglia	Suppress neuroinflammatory cell death	S100A4-mediated PCD pathway	siRNA-mediated S100A4 silencing	Aβ-stimulated BV2 cell model	Transcriptomic and BV2 data support S100A4 involvement; in vivo validation is lacking	[263]
IFN-γ–induced PANoptosis	Suppress Th1-driven epithelial injury	IFN-γ–JAK1 signaling axis	Selective JAK1 inhibitors	Crohn’s disease intestinal organoid model	Enteroid and tissue data support IFN-γ–JAK1-linked PANoptosis	[12]
PD-1/PD-L1 immune-checkpoint biomarker axis	Predict anti-PD-1 treatment outcomes and immune-related adverse events	PD-L1 expression and PD-L1 polymorphisms	Anti-PD-1 antibody therapy with biomarker assessment	Recurrent/metastatic head and neck squamous cell carcinoma	Clinical data support PD-L1 polymorphisms as irAE-related biomarkers	[264]
Inflammatory necroptotic cell death	Suppress cytokine storm and pulmonary inflammation	TLR4–TNF signaling axis	TNF inhibitor etanercept	Influenza A virus infection model	Macrophage and mouse data support TNF-dependent necroptotic inflammation	[265]
Inflammatory PANoptosis	Overcome tumor cell death resistance	TNF-α/IFN-γ and downstream effectors	Combined TNF-α and IFN-γ treatment	Human tumor xenograft models	Cell-line and xenograft data support cytokine-induced PANoptosis	[266]
Sepsis-associated T-apoptosis and immune-checkpoint dysfunction	Stratify sepsis-induced immunosuppression and prognosis	PD-1/PD-L1 axis, CD4+ and CD8 + T-cell depletion	PD-1/PD-L1-axis monitoring; pathway blockade remains a proposed strategy not tested in this cohort	Sepsis and septic shock	Observational sepsis data support PD-1/PD-L1-linked lymphocyte depletion	[267]
ZBP1-mediated inflammatory PCD	Attenuate influenza-associated inflammatory injury	Tripartite motif-containing protein 34 (TRIM34)–ZBP1 ubiquitination axis	Genetic modulation of TRIM34/ZBP1	Influenza A virus infection model	Cell, mouse and patient data support TRIM34–ZBP1 regulation of IAV-induced PCD	[268]
TQ-associated apoptosis/cytotoxicity in HepG2 cells	Induce tumor-cell death and modulate inflammatory signaling	Casp3, DLC1, NF-κB and inflammatory cytokines	Thymoquinone treatment in vitro	HepG2 hepatocellular carcinoma cell model	In vitro data support TQ-induced cytotoxicity and NF-κB suppression	[269]
Apoptosis–autophagy–necroptosis interplay	Regulate inflammation-driven luteolysis	Caspase–ATG–RIPK pathways	Prostaglandin F2α (PGF2α)-induced luteolysis	Bovine corpus luteum tissue and cell models	Review-level evidence supports coordinated PCD in bovine luteolysis	[270]

## Emerging technologies and research

**Fig. 8:**
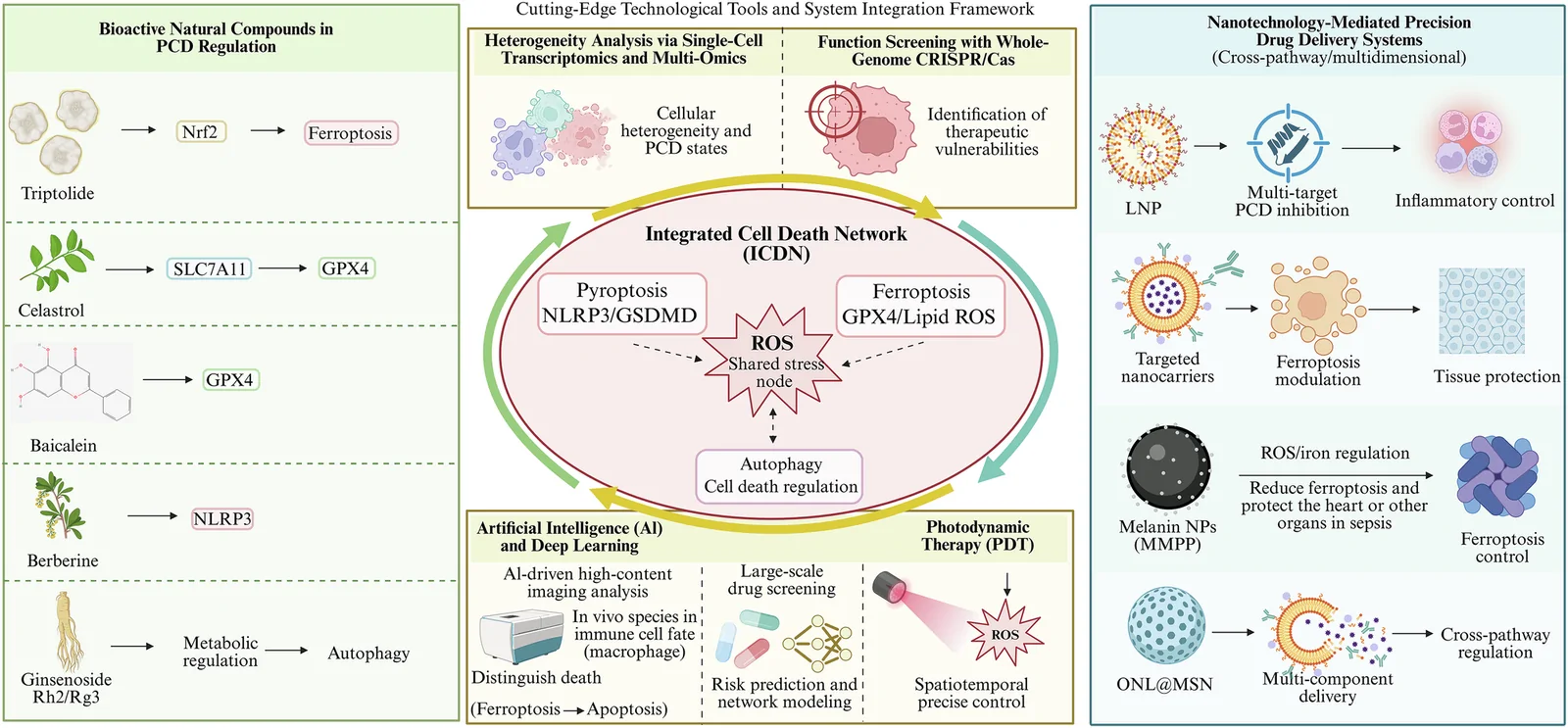
Emerging technologies and intervention strategies for regulating inflammation-associated PCD networks. Bioactive natural compounds have been reported to have multiple regulatory effects on various PCD modalities, such as ferroptosis and pyroptosis, as well as autophagy, through the modulation of key molecular nodes, including those involved in redox homeostasis, inflammasome activation and metabolic signaling pathways. Nanotechnology-based drug delivery systems have been shown to increase drug stability and tissue and cellular targeting, thereby improving therapeutic precision while also permitting coordinated multidrug delivery. These findings provide tools for accurate modulation of PCD signaling cascades in complex inflammatory microenvironments. Moreover, next-generation techniques, including single-cell transcriptomics, integrative multiomics analyses, CRISPR‒Cas-based functional screening, AI-assisted analytics and PDT, provide the ability to elucidate cellular heterogeneity in PCD, identify therapeutic vulnerabilities and realize spatiotemporal control of cell death processes. Overall, these strategies create a cross-pathway, multidimensional research framework that enhances mechanistic insights into inflammation-associated PCD networks and lays the groundwork for precision therapeutic interventions and clinical translation

Because of the rapid development of biotechnology, studies on inflammation-associated PCD have gradually evolved from a pathway-specific approach to a comprehensive model that integrates cross-pathway interactions and multiscale regulation. Instead of specific therapeutic strategies, this section details essential technologies and methodological tools that underpin investigations to date. Natural compounds offer a molecular basis for multitarget adjustments, and nanodelivery systems improve drug stability and targeting in inflammatory microenvironments. More recently, single-cell omics, multiomics integration and clustered regularly interspaced short palindromic repeat (CRISPR)-based screening have permitted high-resolution dissection of the heterogeneity of PCD and its spatiotemporal dynamics. These approaches have thus not only advanced the understanding of inflammation-associated PCD networks but also provided methodological support for identifying key regulatory nodes, tracking dynamic processes, and screening candidate interventions. A representative research framework and application scenario are shown in Fig. 8.

### Applications of bioactive natural compounds in PCD regulation

Over the past few years, these small molecules based on bioactive natural compounds have played a pivotal role in bridging the gap between mechanistic insights and potential regulatory agents. Unlike single-target agents, such molecules generally possess multiple targeting capabilities and, thus, are ideal candidates for investigating synergistic interactions between unique pathways operating within PCD networks. Finally, bioactive natural compounds are referred to in this section not as established therapeutic agents per se but rather as investigational tools and potential modulators to identify regulatory nodes of cell death modalities while their contributions to cell fate modulation are examined [184].

A variety of natural compounds have been used in ferroptosis-related studies to elucidate the connection between redox control and cell death thresholds. For example, triptolide is known to regulate the buildup of lipid peroxidation through antioxidant responses to change cellular susceptibility to oxidative stress [185]. In contrast, celastrol has exhibited contrasting actions in some models, maintaining glutathione metabolism and reducing lipid peroxidation [186]. Such differential regulatory outcomes emphasize the utility of natural products as tools to modulate redox homeostasis and investigate context-specific roles of ferroptosis throughout stages of pathogenesis. In line with these findings, baicalein presents context-dependent regulation of GPX4-associated networks, further confirming that ferroptosis is strongly dictated by cellular and environmental conditions [187].

Apart from ferroptosis, natural products have also shown promising effects on apoptosis and necrosis. Some small molecules are capable of modulating apoptotic processes through changes in mitochondrial function or caspase activity and may also enable cells to transition from apoptosis to other types of death under certain conditions [188]. In relation to necroptosis, certain natural compounds modify inflammatory signaling and cellular stress responses, indirectly affecting RIPK activity and the activation of multiple pathways. Importantly, these studies focus on cell death program plasticity, as opposed to direct inhibition of individual pathways. Natural products have also been employed to elucidate pivotal regulatory nodes involved in pyroptosis, autophagy, and metal-dependent cell death. For example, berberine can reduce inflammasome activity, which in turn promotes the differentiation of metabolic diseases to pyroptosis [189]. By modulating cellular metabolic states, ginsenosides provide experimental models for investigating autophagy-associated cell fate transitions [190]. With respect to emerging modalities such as cuproptosis, research on natural products remains limited; current efforts focus primarily on their roles in mitochondrial metabolism and metal ion homeostasis, indicating substantial room for further exploration.

In summary, the significance of bioactive natural compounds in PCD research lies in their ability to modulate multiple molecular targets. They simulate complex, multipathway changes typical of inflammatory microenvironments, thus aiding in the identification of critical regulatory nodes and pathway cross-talk [191–193]. Future studies should incorporate single-cell omics, multiomics and spatial analyses to systematically compare the action of natural products with their respective effects across different cell types and inflammatory stages and to evaluate their scope of action with regard to specific regulatory nodes and signaling pathways.

### Nanotechnology-mediated drug delivery systems

**Fig. 9:**
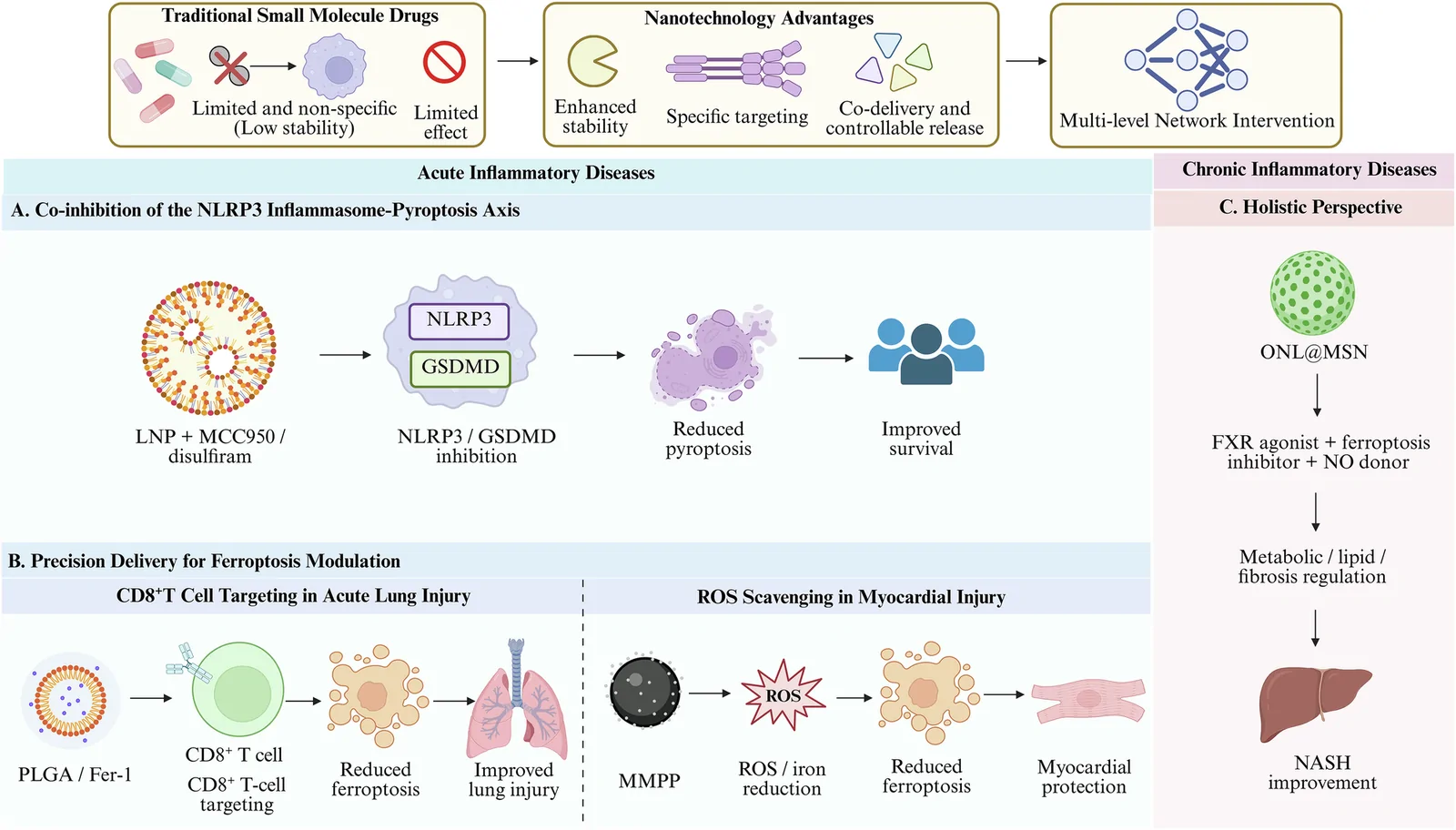
Nanotechnology-mediated drug delivery systems in the regulation of inflammation-associated PCD. **A** Schematic showing that during acute inflammation, LNPs can deliver both the NLRP3 inhibitor MCC950 and the GSDMD inhibitor disulfiram synergistically to inhibit the NLRP3-GSDMD pyroptotic axis. In sepsis models, this alleviates pyroptotic death and enhances survival. **B** Precision delivery strategies for ferroptosis modulators include (i) loading of PLGA nanoparticles with ferrostatin-1 and targeting CD8⁺ T cells to inhibit ferroptosis and ameliorate acute lung injury and (ii) targeting of MMPP, which reduces ROS production and iron overload, thereby inhibiting ferroptosis and alleviating myocardial injury. **C** ONL@MSNs can codeliver an FXR agonist, a ferroptosis inhibitor and an NO donor for chronic inflammatory disease. This system provides multidimensional, cross-pathway control of NASH through the simultaneous control of metabolic homeostasis, lipid peroxidation and fibrosis

As certain approaches converge rapidly across multiple disciplines, nanotechnology-mediated delivery systems have become essential platforms for studying inflammation-associated PCD [194]. In comparison with traditional small-molecule agents, nanocarriers can increase drug stability and biodistribution, whereas surface functionalization allows for enhanced cellular and tissue targeting. These properties drive pathway interrogation and functional assessment in complex inflammatory microenvironments [195]. In addition, coencapsulation of several agents and controlled-release strategies enable the concomitant modulation of multiple signaling nodes and the establishment of an experimental platform for network-level investigations of PCD. Thus far, various nanodelivery systems have been utilized to investigate crucial PCD processes, such as pyroptosis and ferroptosis, as shown in Fig. 9.

In acute inflammatory conditions such as sepsis, pyroptosis is mediated mainly by the NLRP3-caspase-1-GSDMD axis, and its overactivation leads to cytokine storm and multiorgan injury. Codelivery of MCC950 and disulfiram via lipid nanoparticles (LNPs) allows simultaneous perturbation of different inflammasome components. In models of LPS-induced sepsis and peritonitis, this strategy reduces the levels of active caspase-1, IL-1β, and cleaved GSDMD and increases survival [196]. This codelivery system highlights the applicability of such nanoplatforms for the concurrent interrogation of PCD at multiple nodes via dual node targeting.

In addition to pyroptosis, ferroptosis, which is characterized by dysregulated lipid peroxidation and iron accumulation, plays a critical role in various inflammation-associated pathologies. Ferroptosis has been confirmed in acute lung injury and septic cardiomyopathy models with targeted nanodelivery strategies. For example, poly(lactic-co-glycolic acid) (PLGA) nanoparticles coated with a CD8 antibody-modified/PD-1-engineered CD8⁺ T-cell membrane and loaded with ferrostatin-1 allow highly specific targeting of CD8⁺ T cells to assess molecular changes related to ferroptosis in response to LPS-induced acute lung injury [197]. On the other hand, melanin-based nanoparticles (MMPP) that scavenge ROS and inhibit iron deposition have been adopted in sepsis-induced myocardial injury models to understand their interactions with oxidative stress, inflammation, and tissue damage [198]. Overall, these studies not only validate the ability of nanocarriers to improve delivery efficiency but also highlight a tractable system for investigating the crosstalk between oxidative stress, inflammatory signaling outcomes, and PCD.

In chronic inflammatory diseases, their applications as multitarget nanotherapeutics also allow the simultaneous investigation of multiple pathways. In the case of a nonalcoholic steatohepatitis (NASH) model, PEGylated thiolated hollow mesoporous silica nanoparticles that codeliver a farnesoid X receptor (FXR) agonist, a ferroptosis inhibitor, and an NO donor (ONL@MSN) enable the simultaneous evaluation of metabolic homeostasis, lipid peroxidation and fibrotic progression in a single animal model [199]. Such findings support the notion that PCD does not take place in isolation; rather, it is dynamically coordinated with immune modulation, oxidative stress and tissue remodeling.

Nanodelivery systems are useful tools for studying inflammation-associated PCD and have been used successfully as tools for experimental studies investigating pyroptosis, ferroptosis and other processes [194]. However, most studies are limited to preclinical models, and issues with long-term safety, biodistribution stability and clinical translatability remain to be further evaluated. Going forward, greater emphasis on model robustness, in vivo validation and cross-study comparability will help move these platforms toward robust translational interventions.

### Cutting-edge technological tools

PCD research is increasingly moving from single-pathway-specific to cross-scale, systems-level investigative paradigms. Of course, the unparalleled power of these developing modalities is not simply the multidimensionality of data based on high spatial averages derived from a homogeneous population but rather in concretely deconvoluting the cell type-specific, temporal and spatial organization of unique demise programs in bona fide inflammatory microenvironments. Recent advances, such as single-cell omics, AI algorithms and CRISPR-based functional screening, have also enabled research in PCD to evolve beyond descriptive studies into multidimensional analyses involving cellular heterogeneity characterization, identification of key nodes vulnerable to death and tracking of cell death kinetics [200–202].

At the heterogeneity scale, extensive integration of single-cell transcriptomics with bulk omics datasets allows high-resolution characterization of the cell type-specific distribution of pyroptosis-controlled signatures in inflamed tissues or infectious niches. By means of risk stratification models and deep neural network analysis, these features can be associated with clinical parameters, such as disease severity, immune cell infiltration, and therapeutic response, allowing for enhanced systemic characterization of inflammatory states [203]. Concurrently, AI-assisted high-content imaging has greatly enhanced automated phenotyping of cell death modalities to allow precise discrimination between ferroptosis, apoptosis and other types of cell death. This strategy also serves a methodological purpose for enabling large-scale screening of candidate molecules and standardization of cell death phenotypes [204]. Notably, in both infectious and sterile inflammatory contexts, these tools facilitate the identification of dominant PCD modules across spatiotemporal dimensions and their threshold-dependent transitions.

For example, genome-wide CRISPR/Cas functional screening in primary immune cells has been used to systematically identify important regulators of T-cell activation and effector functions [205]. More recently, in vivo pooled CRISPR screening strategies, facilitated by viral delivery and barcode-based lineage tracing, have directly investigated the regulatory dependencies of macrophages, dendritic cells, and T cells on individual cell death pathways in inflammatory or infectious models. These strategies have revealed important molecular nodes that dictate inflammasome activation, gasdermin cleavage, and sensitivity to ferroptosis while also allowing functional validation in the context of native tissue microenvironments [206]. As a spatiotemporally controllable physical induction platform, photodynamic therapy (PDT) can induce lipid peroxidation through exogenous ROS generation, thus modulating ferroptosis-related oxidative stress responses and revealing the structural characteristics of damage thresholds. Emerging evidence suggests that cellular death regulatory strategies, when combined with PDT, provide a methodological framework to reduce or enhance local PCD responses through localized modulation of the intensity of cell death outputs [207, 208].

These advances in the areas of multiomics integration, AI-based analytics and CRISPR functional screening are combined to reshape PCD research around a multidimensional systems framework. They improve the resolution of cellular heterogeneity and dynamic regulation and facilitate the discovery of pivotal regulatory nodes and potential therapeutic targets [209]. However, hurdles lie in cross-platform data integration, model interpretability, and in vivo validation. Future efforts should focus on standardizing data related to PCD, improving cross-model reproducibility, and conducting thorough validation to further elucidate its role in inflammatory diseases. To provide a structured overview of these methodologies and their representative applications for dissecting PCD heterogeneity, identifying regulatory nodes, and enabling downstream experimental design, relevant research strategies and tools are summarized in Table 4.

**Table 4:** Emerging technologies and research directions for dissecting PCD/ICDN networks in inflammatory contexts

Research Area	Technical Approach	Key Methods	Relevance to PCD/ICDN Analysis	Representative Questions	Translational Potential	Current Limitations	References
High-resolution multiomics spatial profiling	Integrated single-cell and spatial multiomics analysis	scRNA-seq, spatial transcriptomics, flow cytometry, immunohistochemistry, coculture assays	Elucidation of inhibin subunit beta A–activin A receptor type 1B (INHBA–ACVR1B) axis–mediated tumor cell apoptosis	How macrophage subsets regulate tumor cell death via specific molecular axes	Targeting the INHBA–ACVR1B axis or macrophage subsets	Limited T-box pituitary transcription factor (TPIT) subtype sample size; insufficient T-cell sequencing depth and clustering resolution; low transcript abundance of key PCD effectors necessitates protein-level and functional validation	[271]
	Integration of spatial metabolomics and transcriptomics	Spatial metabolomics, spatial transcriptomics, lipidomics	G protein-coupled receptor 35 (GPR35)-mediated regulation of lipid droplet formation and cell death	How spatially resolved GPR35 signaling modulates lipid metabolism in MASLD	GPR35 as a therapeutic target in metabolic liver disease	Predominantly correlative; lack of direct functional validation of the GPR35–lipid droplet–cell death axis; limited insight into dynamic mechanisms	[272]
	scRNA-seq	scRNA-seq (10x Genomics)	Profiling apoptosis-associated gene expression changes	Apoptosis and inflammatory transcriptional regulation in pulmonary arterial hypertension endothelial cells	Targeting CD74 to modulate endothelial function	Limited capacity to capture apoptosis per se; constrained by live-cell selection and temporal sampling, potentially underestimating dynamics	[273]
AI and computational biology	Multiomics integration and machine learning modeling	scRNA-seq analysis with machine learning algorithms	Dissecting ICD in bladder cancer	Development of ICD-based prognostic and predictive models	Biomarker discovery and therapeutic stratification	Data heterogeneity compromises model robustness; overfitting and limited interpretability; insufficient clinical validation	[274]
	Multiomics integrative analysis	Machine learning and unsupervised clustering	Association of GALNT6 with pyroptosis pathways	Stratification of inflammatory bowel disease subtypes and biomarker identification	Patient stratification for precision therapy	Limited sample size; high-dimensional low-sample structure increases overfitting risk; trade-off with interpretability	[275]
	Multiomics integration with machine learning	Transcriptomics, single-cell sequencing and ML integration	Mitochondrial dysfunction driving inflammation and neuronal PCD	Mechanistic links between mitochondrial dysfunction and SAE via PCD	Identification of diagnostic biomarkers and early intervention targets	Cross-platform integration affects reproducibility; mechanistic links remain unclear	[276]
High-content spatiotemporal imaging	Live-cell imaging	Microscopy integrated with genetic approaches	Defining morphological and genetic features of T-cell pyroptosis	Reliable discrimination between pyroptosis and apoptosis	Insights into T-cell function in infection and autoimmunity	Technically demanding, low throughput, largely in vitro	[277]
	Live-cell imaging with deubiquitination analysis	Imaging, LDH assay, Western blot, IP-MS, mouse models	GSDME-dependent pyroptosis in radiotherapy	OTUD4-mediated stabilization of GSDME in radiosensitization	Targeting the OTUD4/GSDME axis to enhance radiotherapeutic efficacy in nasopharyngeal carcinoma	The antitumour immune mechanisms mediated by pyroptosis remain incompletely defined; nude mouse models limit assessment of immune effects; and the upstream regulation of the OTUD4/GSDME axis, as well as its interplay with other cell death pathways, remains to be elucidated	[278]
	Live-cell confocal imaging	Transfection, fluorescence staining, time-lapse imaging	Direct visualization of pyroptotic morphology	Gasdermin N-terminal (GSDM-NT) localization and pore formation dynamics	Assessment of GSDM activity and monitoring under pathological conditions	Live-cell imaging mainly captures pyroptotic morphology and lacks independent mechanistic discriminatory power; it provides limited resolution of early events such as inflammasome activation, depends on exogenous expression systems, and remains low throughput	[279]
High-throughput screening and gene editing	Gene editing and molecular interaction analysis	Knockout, scRNA-seq, PLA, Co-IP	Elucidation of the mechanism by which ZBP1 senses Z-DNA to trigger necroptosis	Activation of the necroptotic pathway by mitochondrial Z-DNA via ZBP1	The ZBP1/Zα domain may serve as a therapeutic target for AKI	In vivo evidence is largely based on colocalization and lacks functional validation; the oxalate-induced model has limited generalizability; and the mechanisms underlying Z-DNA formation, together with its interplay with other cell death pathways, remain unclear	[280]
	Gene editing and pharmacological approaches	CRISPR/Cas9 knockout combined with targeted inhibitors	MLKL regulates necroptosis and influences autophagy and parthanatos	Enhancement of HHT-induced cancer cell death by MLKL inactivation	Combination of MLKL inhibitors with HHT for the treatment of CRC	The study is limited to CRC cells and immunodeficient models, restricting generalizability; the mechanism by which MLKL regulates autophagy remains unclear; and the safety and dosing of the combination regimen require further validation	[281]
	FDA-approved drug library screening and integrative multiomics analysis	Phenotypic high-throughput screening, organoid models, mechanistic validation and clinical studies	Investigation of ferroptosis and identification of its specific inhibitors	Inhibition of ferroptosis in pulmonary epithelial cells for the treatment of acute respiratory distress syndrome (ARDS)	Drug repurposing: dipyridamole may represent a potential therapeutic agent for ARDS	The clinical sample size is small, and the underlying mechanism requires further validation	[214]
	Multiomics-guided DC vaccine design and mechanistic analysis	ICD induction, DC coculture, scRNA-seq and in vivo validation	Using ICD to provide DAMPs and antigens to enhance vaccine efficacy	Ineffectiveness of optimized DC vaccines against tumors with T-cell exhaustion	PD-L1 blockade combined with DC vaccination may represent a new therapeutic strategy	The mechanisms by which PD-L1⁺ macrophages suppress T cells remain incompletely understood; the immune effects driven by ICD are still uncertain; and the generalizability of the models is limited	[282]

## Discussion

Most existing strategies for mechanistically parsing PCD are based upon a single-pathway paradigm that fails to account for the integration of diverse cell death modalities within inflammatory microenvironments. PCD has emerged as a significant determinant of the immune response and is directly linked to the initiation, amplification, and resolution of inflammation. Hence, different forms of PCD promote inflammatory responses through the release of DAMPs, modifying cytokine networks and influencing metabolic changes [210–212]. Most importantly, PCD modalities can act through common molecular nodes and signaling platforms to coassemble into an orchestrated regulatory network that may facilitate the context-dependent diversification of inflammatory response cascades [10, 213]. This is where ICDN should be differentiated from PANoptosis. That is, while PANoptosis is a defined conceptual state and form of execution encompassing apoptosis, pyroptosis, and necroptosis, ICDN refers to a broader framework that may involve ferroptotic and cuproptotic pathways, autophagy-associated regulation, ICD, and microenvironment-dependent signaling nodes such as molecules controlling the aforementioned processes. Thus, ICDNs might shed light on why distinct specialized inflammatory diseases show different predominant modalities of cell death, as well as on their respective hub nodes in network structures and therapeutic windows.

Despite the growing understanding of the diverse associations of PCD with inflammation, several important limitations still remain. First, the preponderance of available evidence suggests rather than directly demonstrating dynamic switching between cell death modalities, the involvement of common molecular regulators, and cross-pathway crosstalk. Second, the literature to date remains largely based on one pathway or one cell type, hampering the ability to assess whether network reconfiguration occurs at the in vivo level. Third, most analyses focus on terminal endpoints, and limited in vivo evidence has mapped the spatial redistribution and temporal dynamics of PCD during inflammation [214]. However, at the translational level, the candidate therapies either remain at preclinical stages or perform poorly in relation to genetic diversity in patient cohorts. Even single-pathway blocking interventions are sometimes outflanked by compensatory activation of the PCD network, and multiple pathway strategies face difficulties in terms of safety, dosing optimization, and patient stratification. The lack of clinically actionable PCD-associated biomarkers similarly prevents the evolution of more tailored therapeutic approaches [215]. The inflammatory niche must be thought of as a multicellular and vascular environment rather than an isolated biomatrix component. The influx of proinflammatory macrophages and recruited neutrophils can form a local amplification loop, releasing cytokines, ROS, and damage-associated signals that affect nearby innate or adaptive immune cells as well as nonimmune neighbors. Increased vascular permeability may further promote immune cell access, causing tissue edema and bacterial dissemination that contribute to the establishment of a conducive niche for ICDN activation. As a result, the inflammatory microenvironment illustrated in the conclusion figure should be regarded as a general model contributing to numerous pathological conditions, but its main cellular mediators and types of PCD are disease- and stage specific.

In other words, overcoming these challenges requires moving beyond pathway-centric analyses toward an integrated view of how multiple PCD modalities interact within inflammatory microenvironments. Distinct death programs converge on shared regulatory nodes that coordinate signaling integration and determine cell fate outcomes through competitive and cooperative interactions. For example, RIPK1 and caspase-8 function as key switches controlling the apoptosis‒necroptosis balance, whereas the NLRP3 inflammasome links pyroptosis with broader inflammatory amplification and secondary necrotic signaling. In parallel, GPX4 and FDX1 connect ferroptosis to lipid peroxidation and mitochondrial redox regulation, thereby modulating susceptibility to and interplay with other death pathways under oxidative stress [216]. However, the death modalities and dominant nodes are likely to be context-dependent across infectious, autoimmune, metabolic and tumor-associated inflammation. Studies in the future should expand the application of single-cell and spatial omics to elucidate how such nodes direct the spatiotemporal orchestration and transition of PCD modalities across various cell types and disease stages [217, 218]. Comparative studies across tissues will help in detailing this context-dependent characteristic of dominant death programs and the organization of networks. Targeting shared regulatory nodes such as RIPK1 kinase activity, NLRP3 inflammasome assembly or GPX4-dependent lipid repair systems has generated considerable excitement as a therapeutic concept, as each could coordinate the modulation of multiple interacting PCD outputs. These strategies need to be guided by the optimization of timing, dose and disease context so as not to compromise host defense or physiologic tissue repair. Thus, integrating multiomics data with PCD-related biomarkers in clinical practice will help to stratify patients and assess therapeutic efficacy.

Collectively, the ICDN framework incorporates different PCD modalities within an integrated regulatory framework involving common nodes such as the RIPK1-caspase-8 axis and the NLRP3 inflammasome to coordinate apoptosis, necroptosis and pyroptosis, whereas GPX4-dependent redox control links inflammatory and metabolic signals to ferroptosis [123, 219]. Rather than defining a new cell death modality, ICDN frames how inflammation, metabolism and tissue signals influence the interactive nature of cell death. Such common nodes may also represent potential candidates for intervention; however, the therapeutic importance of these nodes with respect to spatial scale, temporal window, and disease-specific inflammatory contexts has yet to be established.

## Data Availability

Data sharing is not applicable to this article, as no datasets were generated or analyzed during the current study. All the information is derived from publicly available articles and datasets.
